# Cooperative Functionalities in Porous Nanoparticles for Seeking Extracellular DNA and Targeting Pathogenic Biofilms via Photodynamic Therapy

**DOI:** 10.1021/acsami.3c00210

**Published:** 2023-03-09

**Authors:** Hannah Bronner, Fabian Brunswig, Denis Pluta, Yaşar Krysiak, Nadja Bigall, Oliver Plettenburg, Sebastian Polarz

**Affiliations:** † Institute of Inorganic Chemistry, Leibniz-University Hannover, Callinstrasse 9, 30167 Hannover, Germany; ‡ Centre of Biomolecular Drug Research (BMWZ), Institute of Organic Chemistry, Leibniz-University Hannover, Schneiderberg 1b, 30167 Hannover, Germany; § Institute of Medicinal Chemistry (IMC), Helmholtz Centre Munich, Ingolstädter Landstraße 1, D-85764 Neuherberg, Germany; ∥ Institute of Physical Chemistry, Leibniz-University Hannover, Callinstraße 3a, 30167 D-Hannover, Germany; ⊥ Laboratory of Nano- and Quantum Engineering, Leibniz University Hannover, 30167 Hanover, Germany; # Cluster of Excellence PhoenixD (Photonics, Optics and Engineering-Innovation Across Disciplines), Leibniz University Hannover, 30167 Hannover, Germany

**Keywords:** click chemistry, Förster resonance energy transfer (FRET), DNA-binding properties, multifunctional materials, antibacterial photodynamic therapy, biofilm disruption, porous organosilica nanoparticles

## Abstract

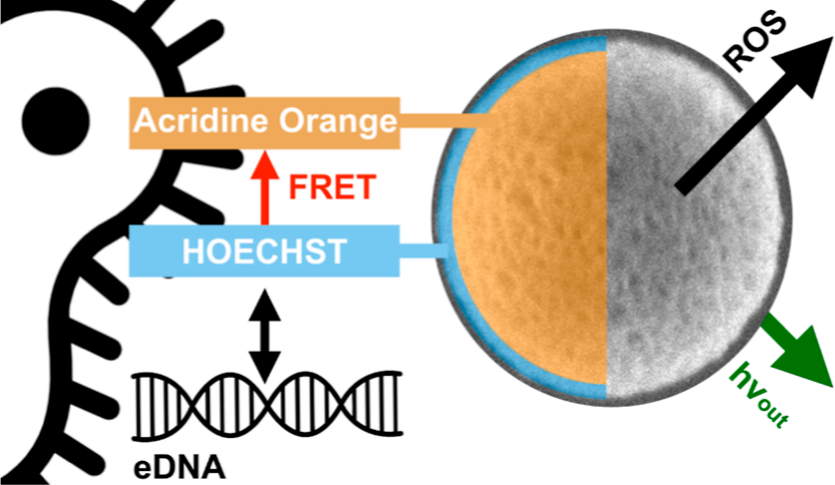

Many pathogenic bacteria are getting more and more resistant against antibiotic treatment and even become up to 1.000× times more resilient in the form of a mature biofilm. Thus, one is currently prospecting for alternative methods for treating microbial infections, and photodynamic therapy is a highly promising approach by creating so-called reactive oxygen species (ROS) produced by a photosensitizer (PS) upon irradiation with light. Unfortunately, the unspecific activity of ROS is also problematic as they are harmful to healthy tissue as well. Notably, one knows that uncontrolled existence of ROS in the body plays a major role in the development of cancer. These arguments create need for advanced theranostic materials which are capable of autonomous targeting and detecting the existence of a biofilm, followed by specific activation to combat the infection. The focus of this contribution is on mesoporous organosilica colloids functionalized by orthogonal and localized click-chemistry methods. The external zone of the particles is modified by a dye of the Hoechst family. The particles readily enter a mature biofilm where adduct formation with extracellular DNA and a resulting change in the fluorescence signal occurs, but they cannot cross cellular membranes such as in healthy tissue. A different dye suitable for photochemical ROS generation, Acridine Orange, is covalently linked to the surfaces of the internal mesopores. The spectral overlap between the emission of Hoechst with the absorption band of Acridine Orange facilitates energy transfer by Förster resonance with up to 88% efficiency. The theranostic properties of the materials including viability studies were investigated in vitro on mature biofilms formed by *Pseudomonas fluorescens* and prove the high efficacy.

## Introduction

Side-effects of conventional treatment of bacterial infections with antibiotics are well known. It can lead to allergic reactions,^1^ heart problems,^2^ sensitivity to light,^3^ and a particular problem is jeopardizing the gastrointestinal tract because antibiotics cannot differentiate between infectious bacterial colonies and healthy intestinal flora.^4^ There is a lack in target specificity and also regarding the site of action. As a consequence, the systemic drug administration is using high dosages for providing a sufficient level of antibiotic concentration. The latter combined with various forms of misuse, e.g., in agriculture has already resulted in multiple and accelerated development of bacterial resistances against antibiotics.^5^ In addition, surface colonization and biofilm formation enable single-cell organisms such as bacteria a multicellular lifestyle which facilitates group behavior and survival in adverse environmental conditions. After initial attachment to a surface, the bacteria produce adhesion proteins and fibers and initiate the production of extracellular polymeric substances (EPS) such as polysaccharides and extracellular DNA (eDNA),^6^ which together act as the ‘glue’ for cell attachment onto the surface and as a stabilizing scaffold for the following biofilm formation. The EPS matrix is protecting the bacteria from various stresses, such as desiccation, predation, oxidizing molecules, radiation, and other damaging agents. For example, surface-associated cells can be up to 1.000-fold more resistant against antibiotic treatments compared to their planktonic single-cell counterparts.

Colonization of surfaces by bacteria is a problem in many areas ranging from food industry, shipping industry, to medicine. The WHO names the failure of antibiotics as one of the biggest threats to global health and predicts a development toward extended hospitalization, rising medical costs, and increased mortality in the foreseeable future.^7^ It turned out that antibiotics and biocides that attack only a single core cellular function are not sufficient to control surface colonization and biofilms. An ideal treatment is not prone to bacterial resistances and targets infected areas only with no effects on healthy tissue. Therefore, it is inevitable to develop novel strategies to combat bacterial settlements.

Impacting a mature biofilm combined with location specificity are only two key features of a desirable, future medication. A theranostic system capable of detecting an infection and inducing treatment at the same time, possibly in an autonomous way, is highly attractive. Therapeutic effects can be activated by internal (e.g., pH-value and enzymes)^8,9^ or external (e.g., ultrasound and light) triggers.^10,11^ Impressive results were presented in the literature on stimuli-responsive drug-delivery materials.^12^ However, a frequent problem that has been encountered is durability. Once the drug is released, the system is exhausted. A promising approach to overcome the mentioned issue is the light-triggered antibacterial photodynamic therapy (aPDT).^13−15^ aPDT is based on a photosensitizer (PS) that upon irradiation with light of the appropriate wavelength produces reactive oxygen species (ROS). ROS are hydroxyl radicals, singlet oxygen, and superoxide anions which cause serious damage to the cell membrane, DNA, and proteins.^13^ Compared to classical treatment with antibiotics, aPDT cannot cause antibacterial resistance.^16^ One class of dyes that is known for its ability to produce ROS are acridines. Next to the use as an anti-tumor agent that can be activated upon ultrasonic irradiation,^17^ Acridine Orange (AO) has just recently been proposed as a novel photosensitizer for photodynamic therapy.^18^ To date, the delivery and precise positioning of the PS is one of the major issues in aPDT. The insufficient uptake of photosensitizers, which are mostly hydrophobic and tend to aggregate, result in too little production of ROS in the region of interest and therefore cannot trigger cell death efficiently.^19^ A possible solution was presented by our group by employing mesoporous organosilica particles (MOPs),^20^ demonstrating the synergistic effect of particles on a surface containing a ROS-producing dye and a nitric-oxide (NO) releasing group. The produced O_2_^–•^ and NO^•^ radicals react, when in spatial proximity to each other, to give the peroxynitrite species NO_3_^–^. The life span of NO_3_^–^ is higher, and it permeates better through the membrane of *Pseudomonas Aeruginosa*, killing it in a quantitative way. Organosilica nanoparticles are used for different nanomedical treatments^21−25^ due to their properties to be non-toxic, non-biodegradable, and easily adjustable in shape and size.^26−29^ The materials can be modified with organic functionalities that allow various chemical conjugation reactions (click-reactions, amide couplings, etc.).^30,31^

**Scheme 1:**
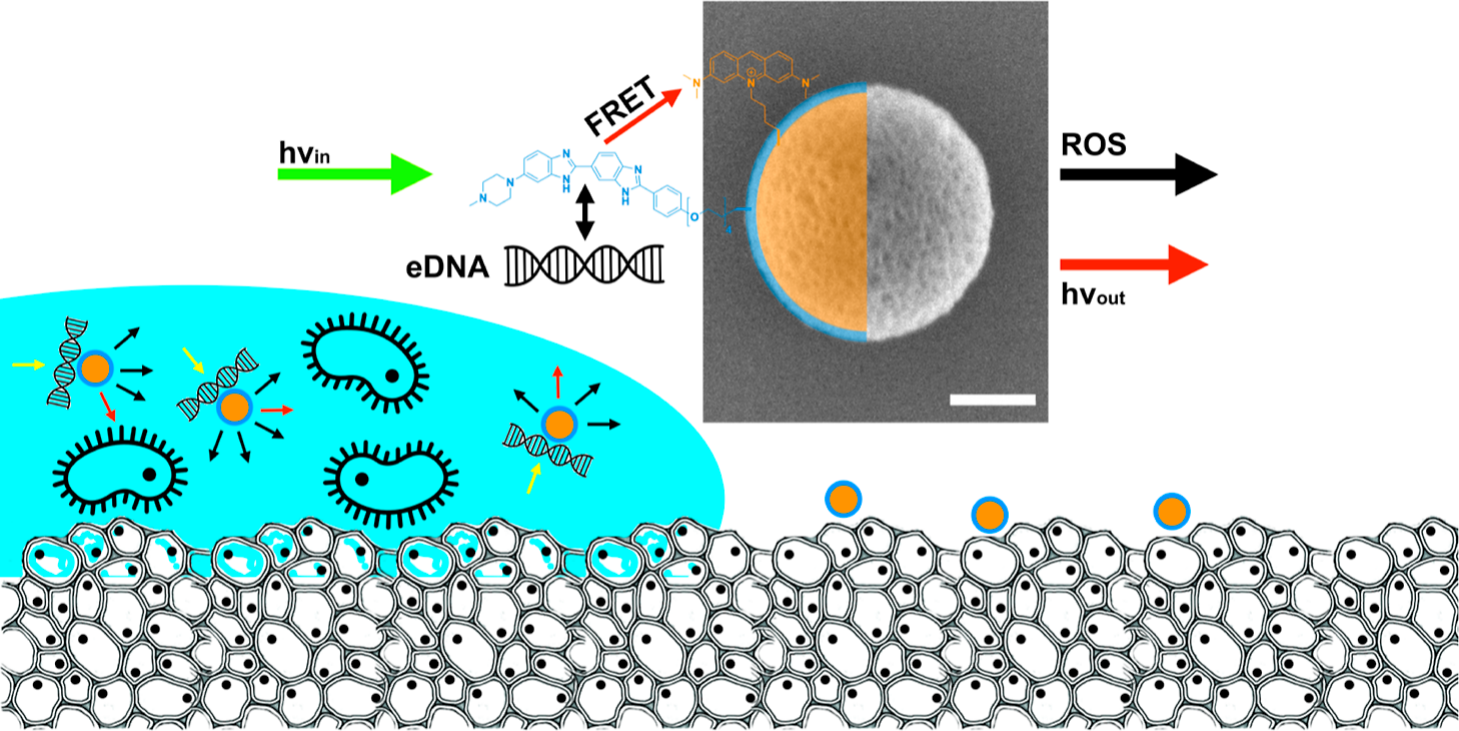
Mesoporous Organosilica Particles (MOPs; SEM Micrograph with Scalebar = 100 nm) In essence, HO-MOPs-AO materials do not only specifically target and fight against a bacterial biofilm (blue); three different functionalities and processes are driven by the exposure to light. The external surface is modified by a special dye (Hoechst; HO), which is capable to specifically form an adduct with extracellular DNA (eDNA) which can be detected by fluorescence (hv_out_). In addition, there is an energy transfer by FRET between the exterior and interior groups. Internal pore surfaces are functionalized with a compound (acridine orange; AO) capable of photodynamic therapy by generation of ROS.

Selective targeting of a bacterial biofilm is a matter of current research, and some impressive progress has been made using nanomaterials.^32−35^ One has learned that the charge of the surface of the nanomaterial is of high importance. Nanoparticles with a natural occurring anionic surface are, without surface modification, a non-ideal system to target a biofilm.^36,37^ For instance, Da Costa et al. could show that a positively charged surface is beneficial for a biofilm affinity, while a negatively charged surface has the opposite effect.^37^ A novel approach is the functionalization of the surface for making it ready to interact with a biological marker in the EPS.^38,39^ Given our experience of the modification of organosilica materials using click-chemistry,^20,40−42^ here, we seek for a possibility for targeting eDNA in the biofilm (see Scheme 1).

An appropriate system is able to bind eDNA, but will not cross the cell membrane where it could bind to genomic DNA. A candidate as a DNA-binding entity is a compound derived from the class of Hoechst molecules.^43^ It is also well known that the binding ability of the molecule is not effected upon functionalization of the binding core.^44^ The manuscript is organized as follows. First, we describe the synthesis and characterization of multifunctional mesoporous organosilica. The second section of the paper discusses the photophysical properties of the material with special emphasis on an energy transfer between the constituents in the material. Finally, we want to probe ROS formation and the theranostic properties in an anti-biofilm application.

## Results and Discussion

### Construction of Multifunctional MOPs

**Scheme 2:**
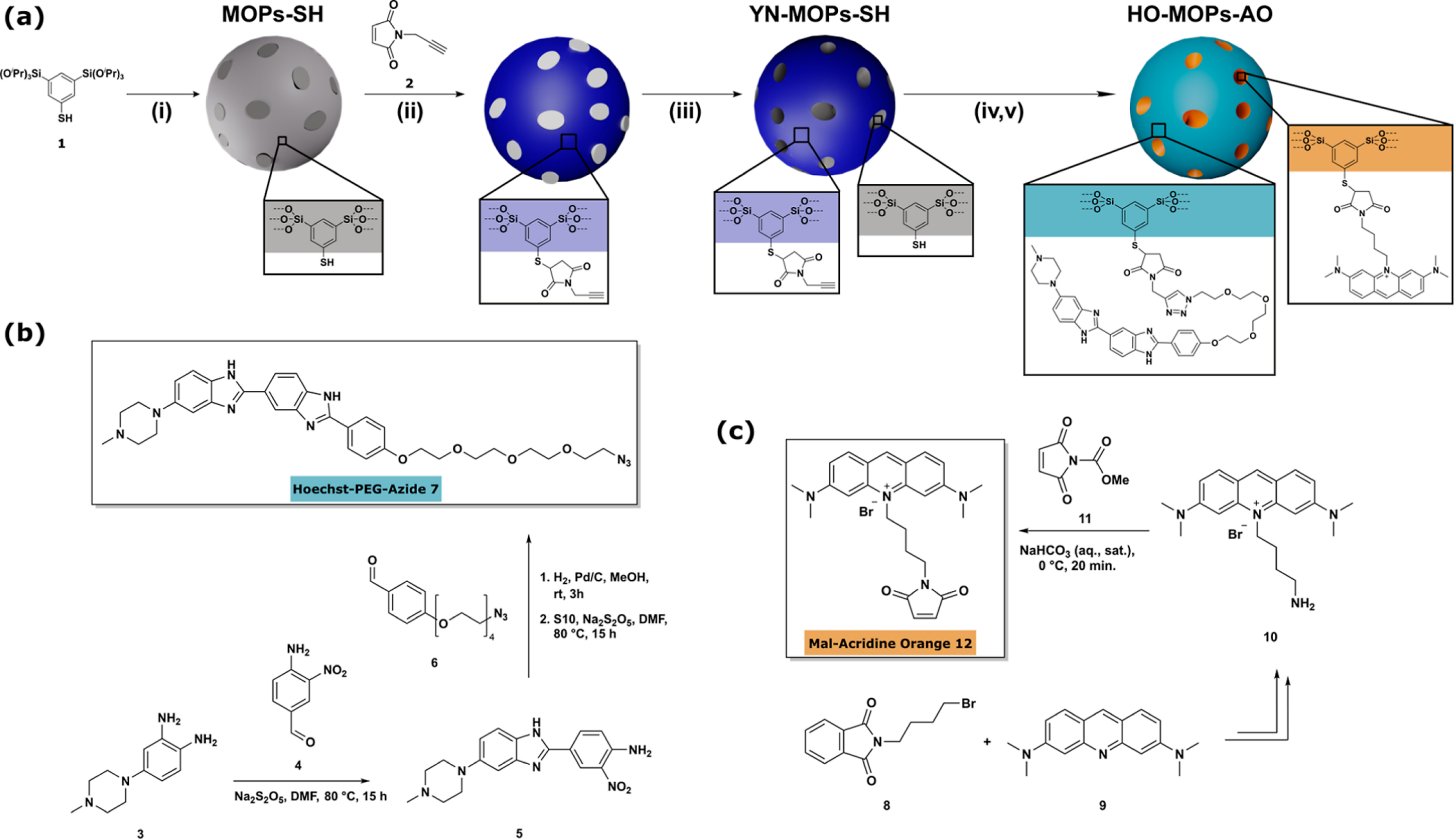
(a) Preparation of Multifunctional Mesoporous Organosilica Nanoparticles (Shown as Spheres) via Spatially and Chemically Orthogonal Click-Chemistry; Step i = Template-Assisted Stoeber Process; Step ii = Modification of the Exterior Surface with Alkyne Groups; Step iii = Template Removal, Step iv = Attachment of (**7**) to the External Surface by Copper-Catalyzed Click Chemistry (Huisgen Cycloaddition); Step v = Attachment of (**12**) by Base-Catalyzed Click Chemistry (Thiol-Michael Addition); (b) Synthetic Pathway Leading to a Click-Ready Derivative of the Hoechst Dye (**7**); (c) Synthetic Pathway Leading to a Click-Ready Derivative of Acridine Orange (**12**)

The desired materials were synthesized, as shown in Scheme 2. Thiol-containing MOPs (MOPs-SH) were prepared by sol–gel chemistry, respectively, by a template-assisted Stoeber process,^45,46^ from 3,5-bis-tri-isopropoxysilyl-thiophenol (**1**) as a precursor (step i; for analytical details, see Figures S5 and S6). For the realization of bifunctionality in a later step, an orthogonal click-chemistry between external and internal surfaces is wanted. The exterior surface can be modified as long as the template molecules have not been removed yet. In agreement to this, there is almost no adsorption seen in N_2_ physisorption measurements (Figure S6e) and the specific surface area is small (17.1 m^2^/g). Because there is no access to the pores of the particles, maleimide click chemistry^47^ can be applied using *N*-propargylmaleimide (**2**; Figure S7) to bring alkyne groups to the external surfaces (see Scheme 2a-ii).

Only after proper extraction of the template, one observes an adsorption isotherm which is characteristic for a mesoporous material (YN-MOPs-SH; see Figure S8). The material has a specific surface area of 907.4 m^2^/g. The presence of the YN and SH groups was checked by spectroscopic methods, e.g., IR and Raman. One can exploit the exterior triple bonds for the copper(I)-catalyzed 1,3 dipolar Huisgen cycloaddition reaction using a suitable azide compound, such as compound (**7**). The synthetic sequence comprising ten steps leading to an azide-modified Hoechst dye with an overall yield of 13% is shown in Scheme 2b and is described in detail in the Supporting Information together with analytical data; see also Figure S9. The subsequent and successful click-addition of (**7**) to the external surfaces (→HO-MOPs-SH) was confirmed via ATR-IR measurements as shown together with additional analytical data in Figure S10. Due to the cycloaddition reaction, two new bands at 1455 and 1612 cm^–1^ appear, which can be assigned to the triazole ring.^48^ The pronounced azide band at 2150 cm^–1^ in the molecular Hoechst-PEG-Azide has disappeared completely which confirms the chemical binding to the nanoparticles.

Finally, the internal surfaces need to be equipped with a photosensitizer for the purpose of aPDT. The accessibility of the internal thiol groups can be proven via the so-called Ellmann essay^49^ (Figure S8c). The preparation of Acridine Orange carrying a maleimide function (**12**) is described in detail in the Supporting Information where additional analytical data is given (Figure S11). After conjugation to the free thiols, we obtain the final material HO-MOPs-AO. The complete characterization of the material is given in Figure S12. In particular, the occurrence of a new band in the IR-spectrum at 1600 cm^–1^ shows the presence of the amine functionalities of the AO rest.

### Photophysical Characterization of HO-MOPs-AO—Diagnostic Functionality

**Figure 1:**
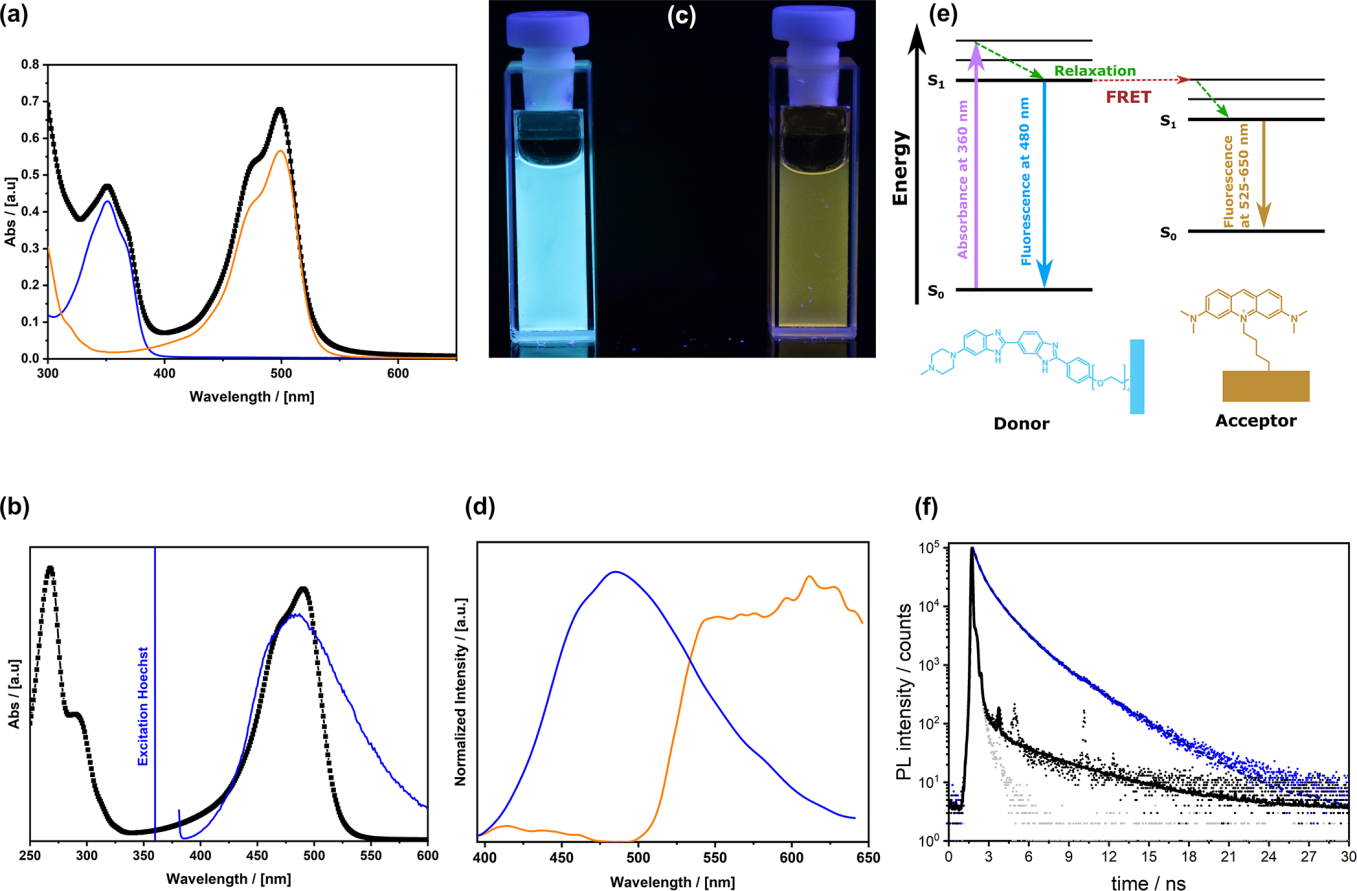
(a) Absorption spectrum of AO (orange), HO (blue), and HO-MOPs-AO (black). (b) Absorption spectrum of AO (black), excitation wavelength, and emission of HO (blue). (c) Photographic image of fluorescence HO-MOPs-SH (left) and HO-MOPs-AO (right) upon irradiation with UV light (365 nm). (d) Emission spectra of HO-MOPs-SH (blue) and HO-MOPs-AO (orange). (e) Schematic Jablonski diagram indicating the FRET process from HO to AO. (f) Fluorescence lifetime measurements of HO-MOPs-SH (blue) compared to HO-MOPs-AO. The instrument response function (IRF) is shown in light gray color.

The absorption properties of the constituents used in the current study are compared to each other in Figure 1a.

One can see that HO-MOPs-AO absorbs at λ_ab,max_ = 351 and 499 nm which correlates to the absorption bands of the HO derivative (**7**) at 350 nm and of the AO (**12**) derivative at 500 nm. Actually, these two compounds have been selected because their spectral features are sufficiently separated from each other and because the fluorescence position of HO fits perfectly to the adsorption band of AO (Figure 1b, see Figure S13 for the absorption and emission spectra of the molecular compounds). Figure 1c shows a photographic image of HO-MOPs-SH excited at 360 nm causing a blue emission at λ_em,max_ = 480 nm (Figure 1d). When AO is present as well (⇒HO-MOPs-AO), the emission of HO seems to be quenched completely and instead one sees a broad signal spanning λ_em_ ≈ 525–650 nm. The data indicate that Förster resonance energy transfer (FRET) is responsible for the observed effect. The Jablonski diagram shown in Figure 1e demonstrates the proposed process between HO as the energy donor (D) and AO as the acceptor (A). In general, FRET occurs not only when there is sufficient spectral overlap, but the spatial distance between “D” and “A” must be smaller than 10 nm.^50^ Because the diameter of the MOPs is ≈300 nm (Scheme 1), one can assume that FRET takes place only between the AO molecules close to the external surface where HO is located. The energy transfer process can be confirmed by fluorescence life-time measurements (Figure 1e). The fluorescence decay curves were fitted with a tri-exponential approximation. The detailed deconvolution fit can be found in the Materials and Methods section, as well as a table with all calculated values (Table S1, Supporting Information). The average lifetime of the excited states in HO-MOPs-SH nanoparticles is 1.387 ns compared to 1.178 ns for HO-MOPs-AO. The shortened lifetime of the donor provides additional evidence that FRET occurs between HO and AO. The FRET efficiency can be calculated as described in the literature (see also the Materials and Methods section) and is surprisingly high (88%).

**Figure 2:**
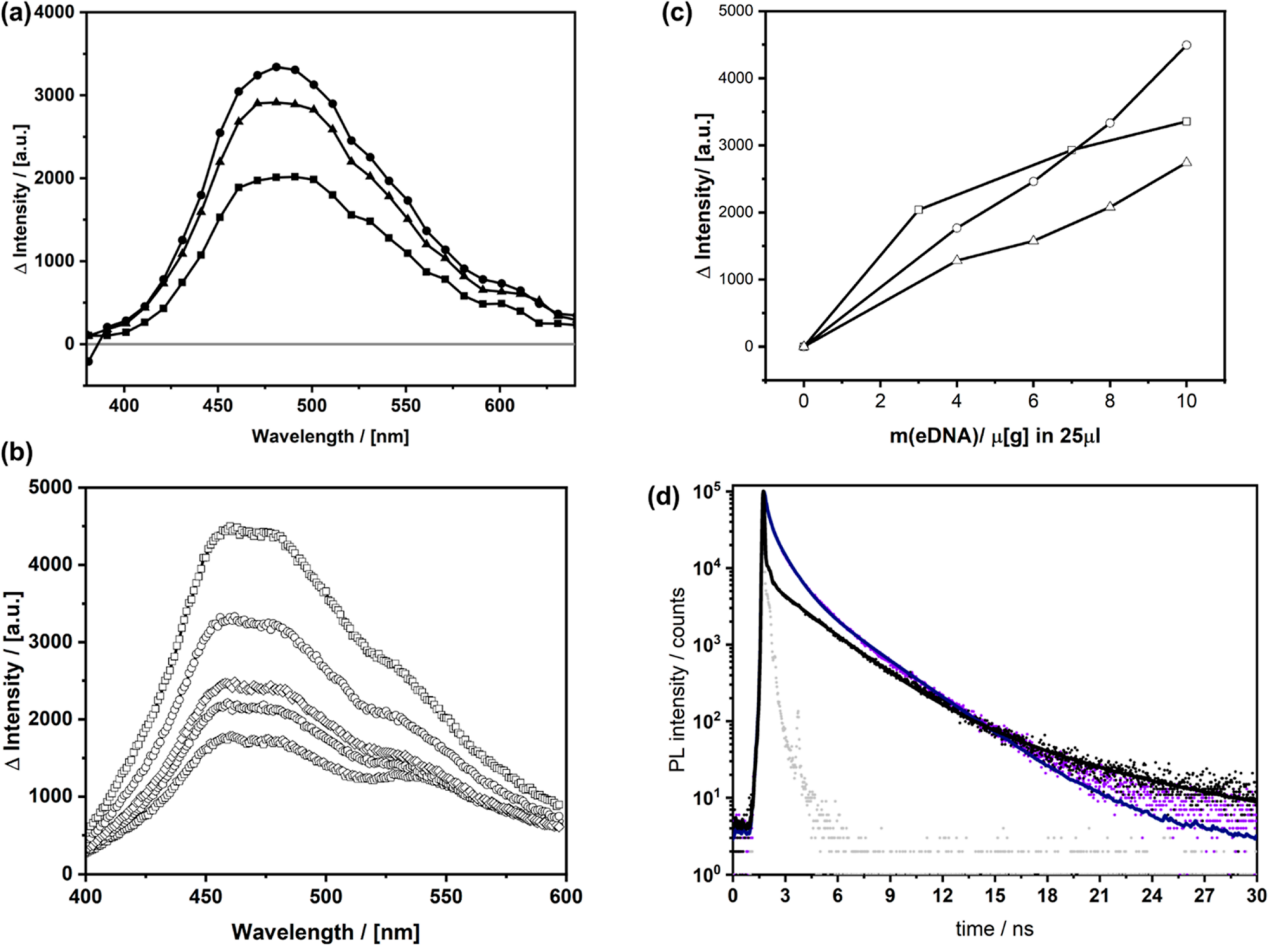
(a) Change of fluorescence induced by the addition of eDNA (0 μg = gray line; 3 μg = black squares; 7 μg = black triangles; 10 μg = black circles) in 25 μL buffer solution to HO-MOPs-SH; excitation at λ_ex_ = 365 nm. (b) Change of fluorescence induced by the addition of eDNA (2 μg = pentagons; 4 μg = hexagons; 6 μg = hashes; 8 μg = circles; 10 μg = squares) in 25 μL buffer solution to HO-MOPs-AO. (c) Change of the fluorescence at λ_em,max_ = 459 nm for HO-MOPs-SH (squares), HO-MOPs-AO (circles), and at λ_em,max_ = 540 nm for HO-MOPs-AO (triangles) as a function of eDNA concentration; excitation at λ_ex_ = 365 nm. (d) Fluorescence lifetime measurements of HO-MOPs-SH (blue) and HO-MOPs-AO in contact with 10 μg eDNA in 25 μL buffer solution (black). The instrument response function (IRF) is shown in light gray color.

The next task is to check if and how the optical properties of the system change caused by a reaction with free DNA molecules. It was mentioned in the introduction section that the Hoechst dye is known for the formation of adducts with DNA leading to a difference in fluorescence. Figure 2a shows that these features are retained also when compound (**7**, Scheme 2) is attached to the exterior surface in HO-MOPs-SH. Relative to the reference state (no DNA⇒Δfluorescence = 0), it can be seen that the intensity at λ_em,max_ = 480 nm becomes higher with increasing DNA concentration (Figure 2a). This behavior is generally similar for HO-MOPs-AO (Figure 2b). However, there are subtle differences. The fluorescence features are more complex.

The fluorescence spectrum seems to be a superposition of 2–3 bands at λ_em,max_ = 459, 480, and 540 nm which we assign to the emission of the DNA/HO complex, HO, and AO. The high energy signals are obviously not anymore quenched completely which is presumably a consequence of the adduct formation with DNA. This means that FRET is not anymore as effective. Nevertheless, some energy transfer still seems to occur because not only the signal at λ_em_ = 459–480 nm depends almost linearly on DNA-concentration, but there is also an increase of the fluorescence associated with AO (Figure 2b). One can see from the fluorescence life-time measurements (Figure 2d) that HO-MOPs-SH and HO-MOPs-AO still behave differently also when DNA is present, but the difference is not as pronounced when there is no DNA (Figure 1f). In line with the arguments given above, the FRET efficiency has decreased to 60%. This effect can be explained as follows. A HO-DNA-interaction can easily affect the distance between HO and AO and can change the intermolecular orientation. Note that the dyes are covalently bound and, thus, immobile. In addition, the complex formation changes the electronic situation in the *π*-system as well.

Comparing the features of HO-MOPs-AO without (Figure 1) and with DNA (Figure 2), one can clearly state that the presence of the intense signal at λ_em_ = 459–480 nm and its concentration dependency is a powerful diagnostic tool. Yet, the capability of HO-MOPs-AO to produce ROS species for antibacterial photodynamic therapy (aPDT) remains an open question.

### Anti-biofilm Activity

**Figure 3:**
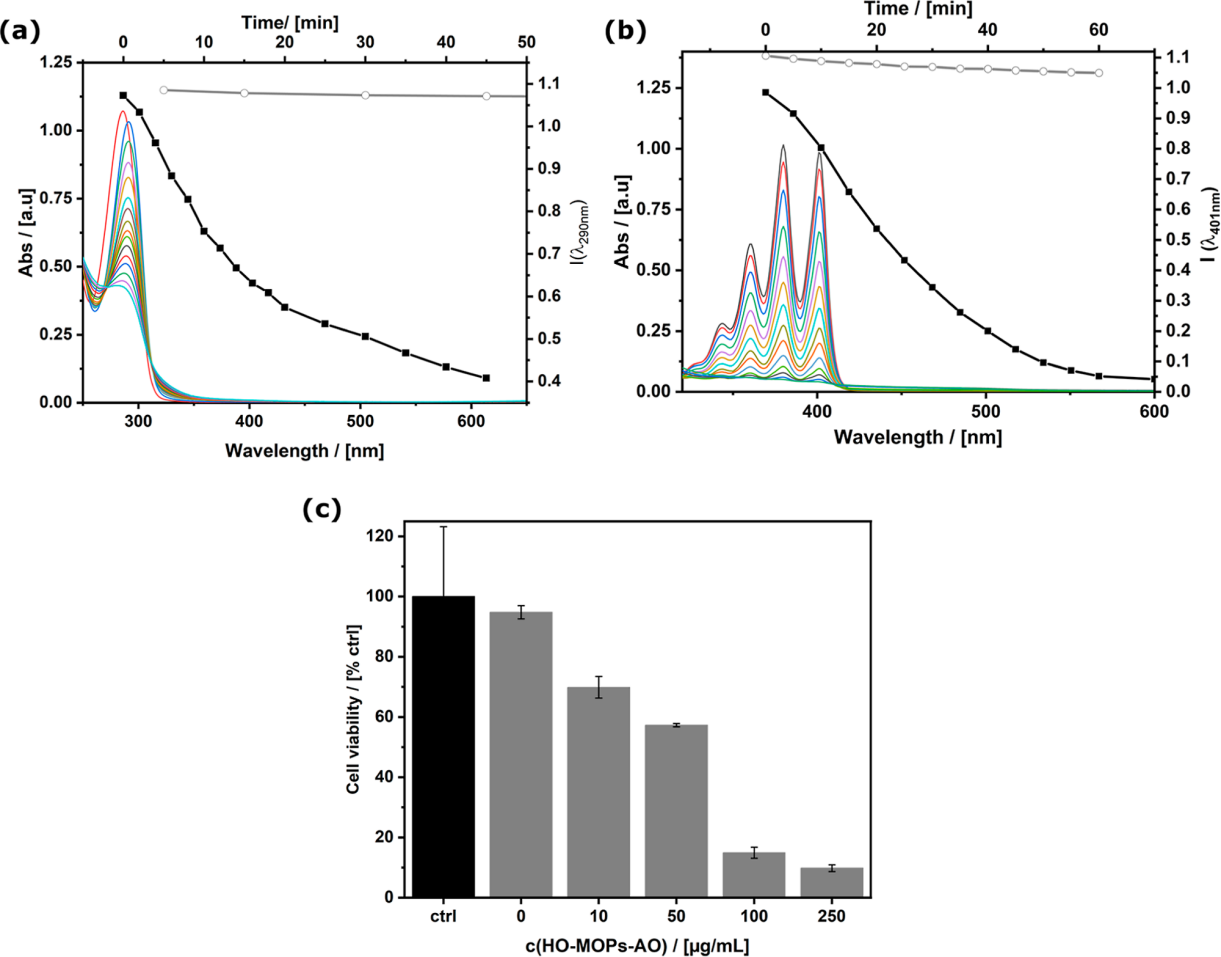
Photo-initiated ROS production by HO-MOPs-AO probed by a (a) uric acid assay; spectroscopic data and time-dependent decay (black squares) of the band at λ_ab_ = 290 nm. As a reference, the result of irradiation of uric acid in the absence of MOPs is shown as well (gray circles; see also Figure S14) and the (b) degradation of 9,10-anthracenediyl-bis(methylene)dimalonic acid (ABDA); spectroscopic data and time-dependent decay (black squares) of the band at λ_ab_ = 401 nm. The results of the irradiation of ABDA in the absence of MOPs is shown as well (gray circles; see also Figure S15b); (c) cell viability after incubation and irradiation of *P. fluorescens* biofilms with increasing amount of HO-MOPs-AO. The black bar represents the control group with no applied material and no light irradiation. The gray bars represent the irradiated biofilms treated with a certain concentration of MOPs. All experiments were performed in triplicates of biologically independent samples. The height of the bars represents the mean of the triplicates, ±SD is indicated.

The capability for ROS production was investigated next. Uric acid is known to scavenge singlet oxygen (^1^O_2_) and is decomposed to triuret, sodium oxanate, allantoxaidin, and carbon dioxide.^51^ Thedecomposition can be followed by a decrease in the absorption band at λ_ab_ = 292 nm as a function of irradiation time. Figure 3a shows the results of the described uric acid degradation assay. It is obvious that only if HO-MOPs-AO is present, ^1^O_2_ is produced and only then decomposition of uric acid occurs. To further investigate and confirm this result, a second literature-known probe^52−54^ was used. 9,10-Anthracenediyl-bis(methylene)dimalonic acid (ABDA) is a water-soluble and anthracene-based probe that reacts with ^1^O_2_ under loss of its condensed aromatic system (the reaction mechanism can be found in Figure S15a, Supporting Information). The absorption spectrum of ABDA which contains three typical absorption bands at 360, 380, and 401 nm does not show any significant change when irradiated without MOPs (see Figure 3b, grey circles). However, when adding 2 mg/mL HO-MOPs-AO and irradiating again, the probe is degraded completely within 70 min (see Figure 3b, black squares). This result is in accordance with the uric acid degradation assay and confirms the production of ^1^O_2_.

**Figure 4:**
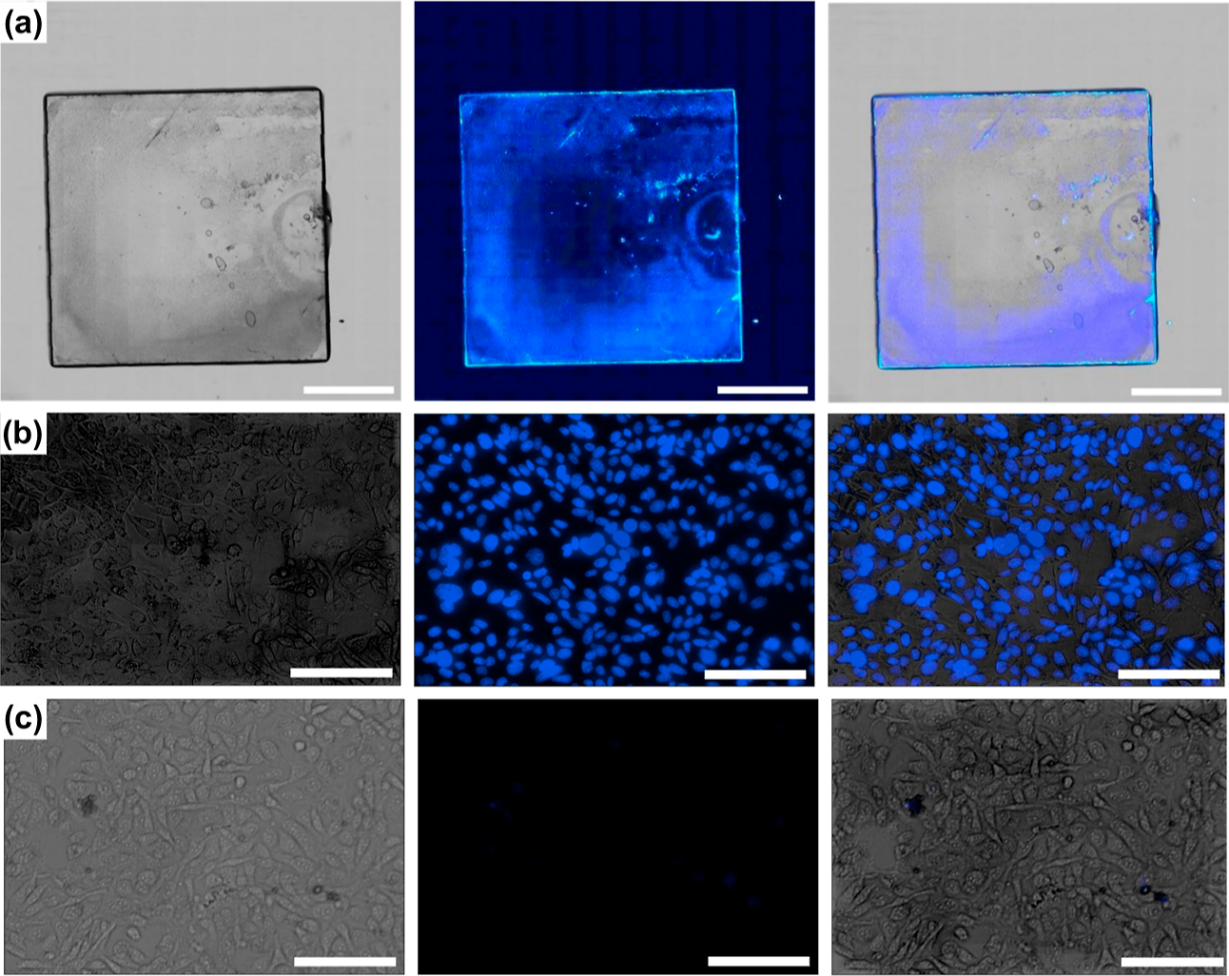
Fluorescence microscopy investigation probing the presence of DNA-HO adduct in MOPs; bright field images (left column); DAPI filter images (middle column); merged channels (right column). (a) *P. fluorescens* biofilm grown on a glass slide; scale bars = 2 mm. *CHO* cells treated with Hoechst 33342 as a reference (b) and treated with MOPs (c); scale bars = 2 mm.

The next task was to study HO-MOPs-AO in contact with a biofilm formed by *Pseudomonas fluorescens* for which external DNA is present (Figure 4). In addition, experiments will be performed with Chinese Hamster Ovary (*CHO*) cells which feature as DNA is solely located in the nuclei. The results will tell if MOPs are unable to cross a cell membrane and thus the interactions with genomic DNA can be avoided. First, it was confirmed that HO-MOPs-AO cannot bind on the bare glass-slide (see Figure S16). Then, a *P. fluorescens* biofilm was cultured on a glass cover slip. The grown biofilm was incubated with HO-MOPs-AO and examined under a fluorescence microscope with a brightfield lamp and a DAPI-filter (Figure 4a). The blue fluorescence signal of HO clearly proves that the particles are inside the biofilm and have reacted with eDNA. As a reference, *CHO* cells were treated with a commercially available Hoechst 33342 with known cell permeability properties next.^55^ As expected, the *CHO* cells exhibit blue fluorescence (Figure 4b). We can now compare to HO-MOPs-AO. From Figure 4c, one sees that HO-MOPs-AO behave different. It cannot enter the *CHO* cells and as a result the fluorescence is absent.

To finish our studies, we performed an incubation/irradiation experiment on a grown biofilm; see Figures 3c and S17. A biofilm was incubated with an increasing amount of particles (0–250 μg/mL) and irradiated with blue light for 180 min. The decrease of living bacteria after this treatment was determined by quantification of the so-called colony forming unit (CFU) in the residual biomaterial (for experimental details, see the Supporting Information). Figure 3c summarizes the results of the antibacterial effect of HO-MOPs-AO (pictures of the agar plates can be found in the Figure S17a). By adding the particles, even the smallest chosen amount (10 μg/mL) led to a decrease of 30% of the living bacteria inside the biofilm. By addition of higher amounts of HO-MOPs-AO, this effect grew to up 90% (cell viability < 10%) with the highest added amount of particles (250 μg/mL). The results obtained for reference experiments are shown in Figure S17b. As expected, there is no effect if the biofilm is exposed to light only and the treatment of the biofilm with HO-MOPs-AO (*c* = 250 μg/mL) in the dark has only a minor effect (cell viability = 90%). Thus, the high activity of HO-MOPs-AO in photodynamic therapy is proven and remarkable as the bacteria in a biofilm are strongly protected against environmental influences and are therefore much harder to combat. With these results, we could show that our system allows a treatment of an biofilm without any pretreatment of the culture dish.^56−58^ Moreover, our system allows a treatment of an already grown biofilm and does not inhibit its formation.

## Conclusions

In the present work, we established a full theranostic tool that allows the detection and the direct treatment of a bacterial infection at its source. We did this by the synthesis of organosilica nanoparticles, which were click-conjugated with two functional units having different purposes on separated positions on the particle. The particles’ surfaces were loaded with a DNA binding Hoechst moiety, the particles’ pores with a ROS producing Acridine Orange photosensitizer. Due to the close distance between the two functionalities and their matching optical properties, we were able to detect FRET between the two dyes. Based on a decreasing FRET efficiency upon DNA addition, we confirmed the interaction of our system with DNA.

By successfully certifying the ability of the particles to bind a bacterial biofilm and not showing any tendency to penetrate a eukaryote’s cell wall, the system enables the detection of biofilms based on fluorescence properties.

To demonstrate the full theranostic character of the system, we verified the particle’s ability to produce ROS upon light irradiation and eventually were able to significantly reduce the number of living bacteria in a model biofilm of *P. fluorescens* by application of our nanoparticles. Due to their theranostic properties, the nanoparticles are also perfectly suited for *in situ* measurements of time-dependent distribution of the nanoparticles inside a biofilm even during the treatment and the interaction between EPS and nanoparticles could be further investigated. This could open new perspectives in the understanding of biofilm dissolution.

The presented system shows promising results in initial *in vitro* experiments and opens a novel perspective for the treatment of superficial infections. The implementation of multi-photon-excitement or IR dyes also opens potential for an application in deeper tissue layers. The observed FRET from the particles’ surface inside its pores has the potential to play a key role, in respect to improve the system toward a broader range of medicinal applications. The FRET could, for example, be used to induce a second reaction, for example, the release of an antimicrobial molecule.

## Materials and Methods

### Preparation of 1,5-Bistri(isopropoxysilyl)-benzene-3-thiol, *N*-Propargylmaleimide, Hoechst-PEG-Azide, and Mal-Acridine Orange

The detailed experimental procedure and molecular analysis of all molecular compounds can be found in the Supporting Information.

### General Synthesis of 1,5-Bistri(isopropoxysilyl)-benzene-3-thiol Nanoparticles^59^

For hydrolysis, 300 mg (0.579 mmol) of 1,5-Bistri(isopropoxysilyl)-benzene-3-thiol is dissolved in 3 mL of isopropanol. 1.86 mL of 0.1 M hydrochloric acid is added while stirring for 3 h. In a second vial 198 mg (0.033 mmol) of Pluronic P123, 120.3 mg (0.33 mmol) of Cetrimonium bromide (CTAB), 310.13 μL (1.29 mmol, 263.61 mg) of triisobutylbenzene (TIB), and 94.56 μL (1.62 mmol, 74.63 mg) of ethanol are dissolved in 6 mL of 0.1 M carbonate buffer (pH = 9) for 3 h. After the complete dissolution of the polymers, the prehydrolyzed 1,5-Bistri(isopropoxysilyl)-benzene-3-thiol solution is quickly added to the buffer and stirred for 2 days. For extraction of the template, the particles are stirred in ethanol: concentrated hydrochloric acid (4:1) for 2 days.

#### Synthesis of Bifunctional 3,5-Bis-tri-isopropoxysilyl-thiophenol Nanoparticles (MOPs-SH)

190 mg (0.895 mmol, 2 equiv) of 1,5-bistri(isopropoxysilyl)-benzene-3-thiol nanoparticles (not extracted) is dispersed in 7 mL phosphate buffer (0.1 M, pH = 8), and 60.44 mg (0.447 mmol, 1 equiv) *N*-Propargylmaleimide is dissolved in 0.5 mL DMSO and added to the nanoparticle solution. The dispersion is stirred for 6 h, and the particles are washed with ethanol and dried. To extract the template, the particles are dispersed in ethanol: hydrochloride acid (4:1), stirred for 2 days, and washed with ethanol and water.

#### Surface Modification of Bifunctional 1,5-Bistri(isopropoxysilyl)-benzene-3-thiol-alkyne (YN-MOPs-SH) Nanoparticles with Hoechst-PEG-Azide (7)

15 mg (0.0706 mmol, 5 equiv) of 1,5-Bistri(isopropoxysilyl)-benzene-3-thiol-alkyne nanoparticles is dispersed in 1 mL of THF/water (1:1, degassed), and 26.3 mg (0.0706 mmol, 5 equiv) tetrakis(acetonitrile)copper(I) hexafluorophosphate and 8.8 mg (0.014 mmol, 1 equiv) Hoechst-PEG-Azide are added to the nanoparticle solution. The dispersion is stirred overnight, and the particles are washed with THF, water, 0.01 M EDTA solution, and ethanol and dried.

#### Pore Modification of Bifunctional 1,5-Bistri(isopropoxysilyl)-benzene-3-thiol-Hoechst (HO-MOPs-SH) Nanoparticles with Modified Acridine Orange (HO-AO)

30.6 mg (0.1441 mmol, 2 equiv) of 1,5-Bistri(isopropoxysilyl)-benzene-3-thiol-alkyne nanoparticles is dispersed in 2 mL aq. NaHCO3 (0.1 M), and 30 mg (0.0719 mmol, 1 equiv) of Mal-Acridine Orange is added to the nanoparticle suspension. The dispersion is stirred overnight, and the particles are washed with MeOH, water, and ethanol and dried.

#### Analysis of the DNA Binding Ability, ROS Production, Biofilm Binding, and Antibacterial Effect of the Functionalized Nanoparticles

Detailed description of the analytical methods and biofilm preparation can be found in the Supporting Information.

## Supporting Information Available

Experimental details and additional data (PDF)

## Notes

All data are available in the main text or the Supporting Information.
