# Supplementary material for: Cooperative Functionalities in Porous Nanoparticles for Seeking Extracellular DNA and Targeting Pathogenic Biofilms via Photodynamic Therapy
Source: ACS Appl Mater Interfaces. 2023 Mar 9;15(11):14067–76. doi: 10.1021/acsami.3c00210 (PMC10037239; doi:10.1021/acsami.3c00210)
Supplement: Supplementary file 1 — am3c00210_si_001.pdf [file am3c00210_si_001.pdf]

Supporting Information

Cooperative Functionalities in Porous Nanoparticles for Seeking Extracellular DNA and Targeting Pathogenic Biofilms via Photodynamic Therapy

Hannah Bronner,[a]‡ Fabian Brunswig,[b,c]‡ Denis Pluta,[d,e,f] Yaşar Krysiak,[a] Nadja Bigall,[d,e,f] Oliver Plettenburg[b,c,e,g] and Sebastian Polarz*[a,e,f]*

[a] Institute of Inorganic Chemistry, Leibniz University Hannover, Callinstrasse 9, 30167 Hannover, Germany

[b] Centre of Biomolecular Drug Research (BMWZ), Institute of Organic Chemistry, Leibniz-University Hannover, Schneiderberg 1b, 30167 Hannover, Germany

[c] Institute of Medicinal Chemistry (IMC), Molecular Targets and Therapeutics Center, Helmholtz Center Munich, D-85964 Neuherberg, Germany

[d] Institute of Physical Chemistry, Callinstraße 3a, 30167 D-Hannover, Germany

[e] Laboratory of Nano- and Quantum Engineering, Leibniz University Hannover, 30167 Hanover, Germany

[f] Cluster of Excellence PhoenixD (Photonics, Optics and Engineering -Innovation Across Disciplines), Leibniz University Hannover, 30167 Hannover, Germany

[g] Institute of Lung Health (ILH), 35392 Gießen, Germany

Email: Oliver.plettenburg@aca.uni-hannover.de, Sebastian.polarz@aca.uni-hannover.de

SUPPORTING INFORMATION

Table of Contents

Experimental procedures	S3
Analytical methods	S12
Supporting Figures	S19
References	S63

Experimental Procedures

Chemicals were purchased from Sigma-Aldrich and TCI Chemicals. SEM images were obtained using a JSM-6700F (JEOL Ltd., Akishima, Japan) and a (Hitachi Ltd., Chiyoda, Japan). EDX measurements were EDX Oxford Ultim Max $100mm^2$ windowless. TEM images were taken with a HT7800 (Hitachi Ltd., Chiyoda, Japan). UV-Vis measurements were carried out using a Cary 5000 spectrometer (Agilent Technologies Inc., Santa Clara, CA, USA). FT-IR measurements were obtained with a Tensor 27 FTIR (Bruker, Billerica, MA, USA). DLS measurements were obtained with a Zetasizer ZMV2000 (Malvern, Kassel, Germany). N2-physisorption measurements were recorded on a Micromeritics Tristar. NMR measurements were acquired on a Ultrashield 400 MHZ (ULS400) (Bruker, Billerica, MA, USA). Fluorescence measurements were carried out with a Cytation 5 (Agilent Technologies, Santa Clara, California, USA). High resolution mass spectrometry (HRMS) was performed at a Micromass LCT-Premier (Waters, Milford, MA, USA) spectrometer using a Lockspray Dual Ion Source and a Waters Alliance 2695-System. Ionization took place via electrospray ionization (ESI). All values are stated in mass/charge (m/z). LC-MS data as reaction controls was recorded at an 1100 series HPLC system (Agilent Technologies, Santa Clara, California, USA) coupled with an Esquire 3000plus MS detector (Bruker, Billerica, MA, USA) using C8-HPLC cartridges.

Molecular synthesis

Synthesis of 1,5-Bistri(isopropoxysilyl)-benzene-3-thiol (1). [1]To a solution of 6 g of 1,3-bistri(isopropoxy)silyl-5-bromobenze (10.6 mmol) in 400 mL of dry Et_2O was added ^{t}BuLi (11.5 mL, 1.9 M, 21.2 mmol) dropwise at - 78 °C. The mixture was stirred for 30 min. Then 355 mg (10.6 mmol) of S_8 was added and stirred for another 30 min at – 78 °C. Afterwards the colorless

solution was warmed to room temperature and stirred for 1.5 h. Then the reaction was hydrolyzed with 30 mL of dry Isopropanol. After removal of the solvent a yellow oil can be obtained. For further purification column chromatography was applied (silica gel 60, CH_2Cl_2). Finally, 5.38 g (10.37 mmol; >95 %) of a colorless oil was obtained. **^{1}H-NMR (400 MHz, $CDCl_3$):** δ / [ppm] 1.20 (d, 36 H, ^{i}Pr-CH_3), 3.42 (s, 1H, SH), 4.24 (sept, 6H, ^{i}Pr-CH), 7.62 (s, 2 H, o-arom. H), 7.76 (s, 1H, p-arom. H)

Synthesis of *N*-Propargylmaleimide (2).[2] 1.1 g (20 mmol, 1 eq) propargylamine and 1.96 g (20 mmol, 1 eq) maleic anhydride are dissolved in 40 mL of acetic acid and stirred overnight in the dark. The solvent was evaporated and the crude solid was suspended in acetic anhydride (18 mL). The suspension is stirred for 2 hours at 65 °C, cooled down to room temperature and poured into ice-cold water. The mixture is extracted with diethyl ether (3 x 100 mL), the organic phase dried over $MgSO_4$ and evaporated. The crude product is purified by silica flash column chromatography (pentane: ethyl ether 6:1 to 4:1) to result an off-white solid. **^{1}H-NMR: (400 MHz, $CDCl_3$):** δ (ppm) = 6.76 (s, 2 H), 4.30 (d, 2 H), 2.21 (t, 1 H).

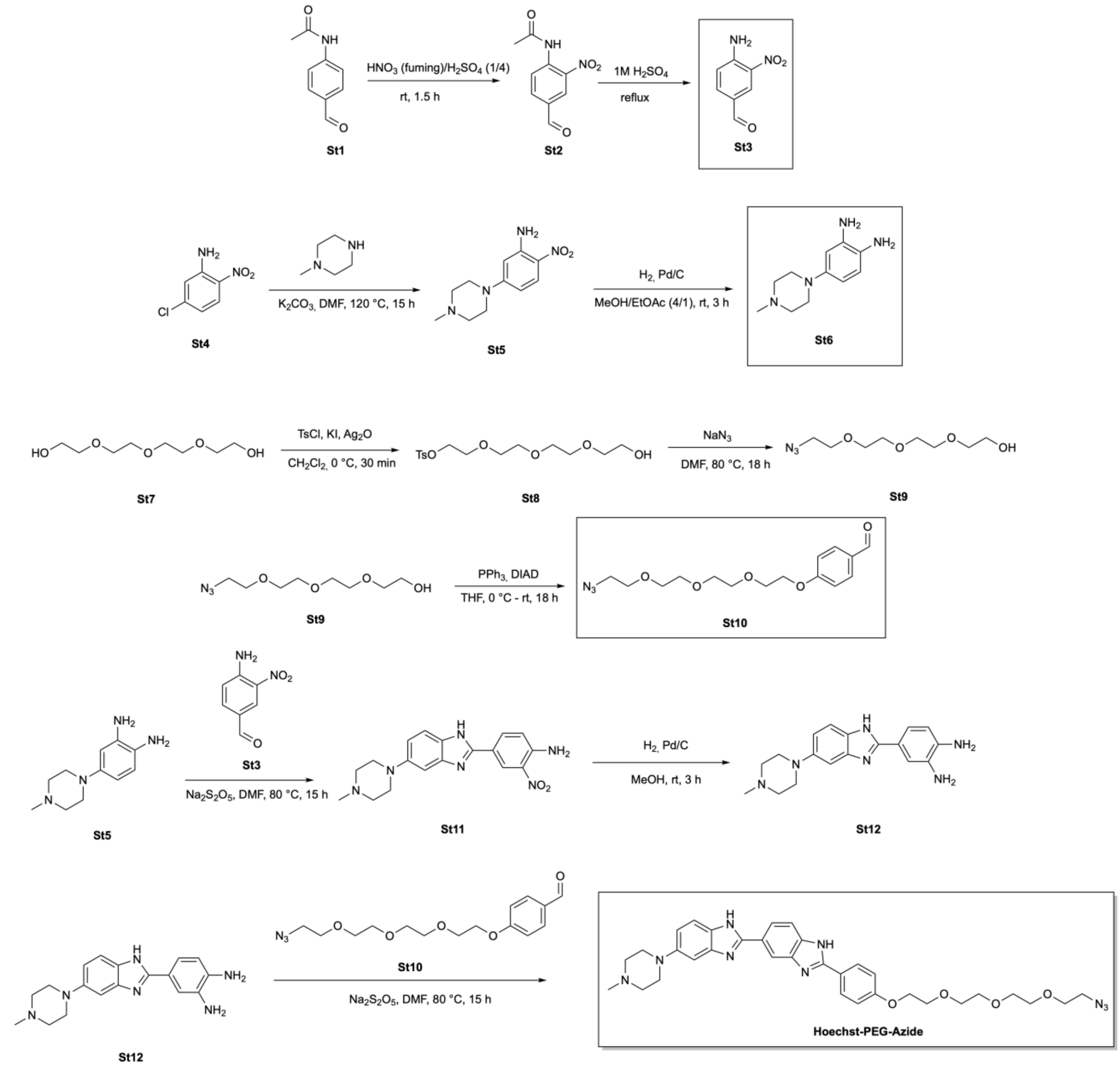

Figure S1: Synthesis of Hoechst-PEG-Azide/ compound (**7**)

Preparation of St2. HNO_3 (fuming, 700 µl) was added dropwise to conc. H_2SO_4 (2800 µl) at 0 °C. S1 (1000 mg, 6.10 mmol) was added to this solution in portions. After complete addition the reaction was stirred for 30 min and after completion, the reaction was quenched by addition of sat. Na_2CO_3 solution. The mixture was extracted with CH_2Cl_2. The combined organic phases were dried over Na_2SO_4, filtered and the solvent was removed under reduced pressure. The crude product was purified over silica (PE/EA = 3/1 – 1/1). St2 (1071 mg, 5.21 mmol, 84 %) was isolated

as a yellow solid. **^{1}H NMR (400 MHz, $CDCl_3$)** δ = 10.66 (s, 1H), 10.01 (s, 1H), 9.06 (d, J = 8.8 Hz, 1H), 8.77 (d, J = 2.0 Hz, 1H), 8.18 (dd, J = 8.8 Hz, 2.0 Hz, 1H), 2.38 (s, 3H). The analytic data correspond to reporting literature.[3]

Preparation of St3: St2 (1990 mg, 9.60 mmol) were suspended in H_2SO_4 (10 vol.%, 9.5 ml) and the suspension was stirred for 1.5 h at 100 °C. After completion, the reaction was quenched by addition of sat. Na_2CO_3 solution. The mixture was extracted with CH_2Cl_2. The combined organic phases were dried over Na_2SO_4, filtered and the solvent was removed under reduced pressure. St3 (1.52 g, 9.1 mmol, 96 %) was isolated as a yellow soild and was used without further purification. **^{1}H NMR (400 MHz, DMSO) δ** = 9.76 (s, 1H), 8.57 (d, J = 2.0 Hz, 1H), 8.25 (bs, 2H), 7.80 (dd, J = 8.9, 2.0 Hz, 1H), 7.12 (d, J = 8.8 Hz, 1H). The analytic data correspond to reporting literature.[4]

Preparation of St5: 5-chloro-2-nitroaniline (S4) (250 mg, 1.5 mmol), K_2CO_3 (300 mg, 2.2 mmol) and *N*-Methylpiperzine (193 µl, 1.7 mmol) were suspended in DMF (870 µl) and stirred for 15 h at 120 °C. After this time, the solvent was removed under reduced pressure and the residue was taken up in water and CH_2Cl_2. The aqueous phase was extracted with CH_2Cl_2, the combined organic phases were dried over Na_2SO_4, filtered and the solvent was removed under reduced pressure. The crude product was purified over silica (CH_3OH in CH_2Cl_2: 1 % - 6% - 10 %). St5 (287 mg, 1.2 mmol, 84 %) was isolate as a yellow sloid. **^{1}H NMR (400 MHz, $CDCl_3$):** δ = 8.03 (d, J = 9.8, 1H), 6.30 (dd, J = 9.7 Hz, 2.6 Hz, 1H), 6.17 (s, 2H), 5.97 (d, J = 2.6 Hz, 1H), 3.39 (t, J = 5.1 Hz, 4H), 2.54 (t, J = 5.1 Hz, 4H), 2.36 (s, 3H). The analytic data correspond to reporting literature.[5]

Preparation of St6: St5 (192 mg, 0.81 mmol) was dissolved in EtOAc/CH_3OH (4/1, 7.5 ml). Palladium on activated charcoal (10 %, 10 mg) was added to this solution and hydrogen gas was bubbled through this suspension for 2 h. After this time, the mixture was filtered over celite and the solvents were removed under reduced pressure. St6 (163 mg, quant.) was isolated as a brown oil and used directly in subsequent steps, in expectance of instability of the product.

Preparation of St8: St7 (1.99 g, 10.3 mmol) was dissolved in CH_2Cl_2 (7 ml) at 0 °C. *p*-toluenesulfonylchloride (2.15 g, 11.3 mmol), Ag_2O (3.6 g, 15.4 mmol) and KI (340 mg, 2.05 mmol) were added to the solution and the resulting suspension was stirred for 20 min at this temperature. After this time the suspension was filtered over Celite and the solvent was removed under reduced pressure. The crude material was purified over silica (PE/EA = 1/1 – 1/3 – 0/100). St8 (2.2 g, 6.5 mmol, 63 %) was isolated as a colorless oil. **^{1}H NMR (400 MHz, $CDCl_3$):** δ = 7.78 (d, J = 8.3 Hz, 2H), 7.32 (d, J = 7.9 Hz, 2H), 4.16 – 4.13 (m, 2H), 3.69 – 3.57 (m, 14H), 2.43 (s, 3H). The analytic data correspond to reporting literature.[6]

Preparation of St9: St8 (1.2 g, 3.5 mmol) was dissolved in DMF (7 ml). NaN_3 (560 mg, 8.6 mmol) was added to this solution and the resulting suspension was stirred for 15 h at 80 °C. After this time, the solvent was removed under reduced pressure and the residue was taken up in water and CH_2Cl_2. The aqueous phase was extracted with CH_2Cl_2 and the combined organic phases were washed with sat. NaCl solution once. The organic phase was dried over Na_2SO_4, filtered and the solvent was removed under reduced pressure. St9 (734 mg, 3.3 mmol, 97 %) was isolated as a colorless oil and was used without further purification. **^{1}H NMR (400 MHz, $CDCl_3$):** δ = 3.80 – 3.73 (m, 1H), 3.72 – 3.68 (m, 5H), 3.66 – 3.61 (m, 1H), 3.42 (t, J = 5.1 Hz, 1H). The analytic data correspond to reporting literature.[7]

Preparation of St10: St9 (1257 mg, 5.7 mmol), 4-Hydroxybenzaldehyd (910 mg, 7.5 mmol) and triphenylphosphine (1298 mg, 7.5 mmol) were dissolved in THF (30 ml) at 0 °C. Diisopropyl azodicarboxylate (1.2 ml, 7.5 mmol) was added dropwise and after complete addition, the reaction was stirred for 18 h and was allowed to warm to room temperature. After completion, the solvent was removed under reduced pressure. The residue was taken up in water and CH_2Cl_2. The aqueous phase was extracted with CH_2Cl_2. The combined organic phases were dried over Na_2SO_4, filtered and the solvent was removed under reduced pressure. The crude product was purified over silica (PE/EA = 3/1 – 1/1). St10 (1588 mg, 4.9 mmol, 83 %) was obtained as a colorless oil. **^{1}H NMR (400 MHz, $CDCl_3$)** δ = 9.85 (s, 1H), 7.79 (d, *J* = 8.8 Hz, 2H), 6.99 (d, *J* = 8.8 Hz, 2H), 4.26 – 4.08 (m, 2H), 3.93 – 3.83 (m, 2H), 3.78 – 3.58 (m, 10H), 3.34 (t, *J* = 5.0 Hz, 2H). **^{13}C NMR (400 MHz, $CDCl_3$)** δ 190.8, 163.8, 131.9, 130.0, 114.8, 70.8, 70.7, 70.6, 70.6, 70.0, 69.4, 67.7, 50.6. R_f (PE/EA: 1/1) = 0.25. **HRMS (ESI):** *m*/*z* calculated for $C_{15}H_{22}N_3O_5$ $[M+H]^+$ = 324.1559; found: 324.1561.

Preparation of St11: St3 (390 mg, 2.35 mmol), St6 (508 mg, 2.47 mmol) and $Na_2S_2O_5$ (446 mg, 2.35 mmol) were suspended in DMF (4.6 ml). The resulting suspension was stirred for 15 h at 80 °C. After this time, the solvent was removed under reduced pressure and the crude product was purified over silica (10 - 12 % CH_3OH in CH_2Cl_2). St11 (437 mg, 1.24 mmol, 53 %) was isolated as an orange solid

^{1}H NMR (400 MHz, DMSO-d_6): δ = 12.65 (s, 1H), 8.76 (s, 1H), 8.15 (dd, *J* = 8.9 Hz, 2.1 Hz, 1H), 7.78 (s, 2H), 7.43 (s, 1H), 7.15 (d, *J* = 8.9 Hz, 1H), 6.93 (d, *J* = 9.0 Hz, 1H), 3.17 (s, 4H), 2.64 (s, 4H), 2.34 (s, 3H). **^{13}C NMR (101 MHz, DMSO):** δ 148.0, 147.2, 141.4, 136.6, 133.8, 130.5, 123.3, 120.3, 118.5, 54.9, 49.8, 49.0, 46.0, 45.5. R_f (12 % CH_3OH in CH_2Cl_2) = 0.15. **HRMS (ESI):** *m*/*z* calculated for $C_{18}H_{21}N_6O_2$ $[M+H]^+$ = 353.1726; found: 353.1722.

Preparation of St12: St11 (379 mg, 1.08 mmol) was dissolved in CH_3OH (2.5 ml). Palladium on activated charcoal (10 %, 10 mg) was added to this solution and hydrogen gas was bubbled through this suspension for 1 h. After this time, the mixture was filtered over celite and the solvents were removed under reduced pressure. St12 (346 mg, quant.) was isolated as a brown oil and used directly in subsequent steps, assuming the instability of the product.

Preparation of Hoechst-PEG-Azide (7): St12 (346 mg, 1.08 mmol), St10 (350 mg, 1.08 mmol) and $Na_2S_2O_5$ (206 mg, 1.08 mmol) were suspended in DMF. The resulting suspension was stirred for 15 h at 80 °C. After this time, the solvent was removed under reduced pressure and the crude product was purified over silica (10 – 15 % CH_3OH in CH_2Cl_2). (7) (498 mg, 0,80 mmol, 74 %) was obtained as a yellow solid.

^{1}H NMR (400 MHz, MeOD) δ = 8.27 (s, 1H), 8.06 (d, *J* = 8.8 Hz, 2H), 7.96 (dd, *J* = 8.5 Hz, 1.7, 1H), 7.70 (d, *J* = 8.5 Hz, 1H), 7.53 (d, *J* = 8.8 Hz, 1H), 7.16 (d, *J* = 2.2 Hz, 1H), 7.11 (d, *J* = 8.9 Hz, 2H), 7.07 (dd, *J* = 8.8 Hz, 2.2, 1H), 4.25 – 4.16 (m, 2H), 3.92 – 3.86 (m, 2H), 3.76 – 3.62 (m, 10H), 3.41 – 3.34 (m, 2H), 3.28 (t, *J* = 5.1 Hz, 5H), 2.82 (t, *J* = 5.0 Hz, 4H), 2.49 (s, 3H).

^{13}C NMR (101 MHz, MeOD) δ 161.1, 153.9, 152.4, 148.0, 128.2, 124.3, 121.7, 121.1, 115.1, 114.8, 70.4, 70.3, 70.3, 70.1, 69.7, 69.3, 67.4, 54.6, 50.4, 50.1, 44.3. R_f (20 % CH_3OH in CH_2Cl_2) = 0.15.

HRMS (ESI): *m*/*z* calculated for $C_{33}H_{40}N_9O_4$ $[M+H]^+$ = 626.3203; found: 626.3205

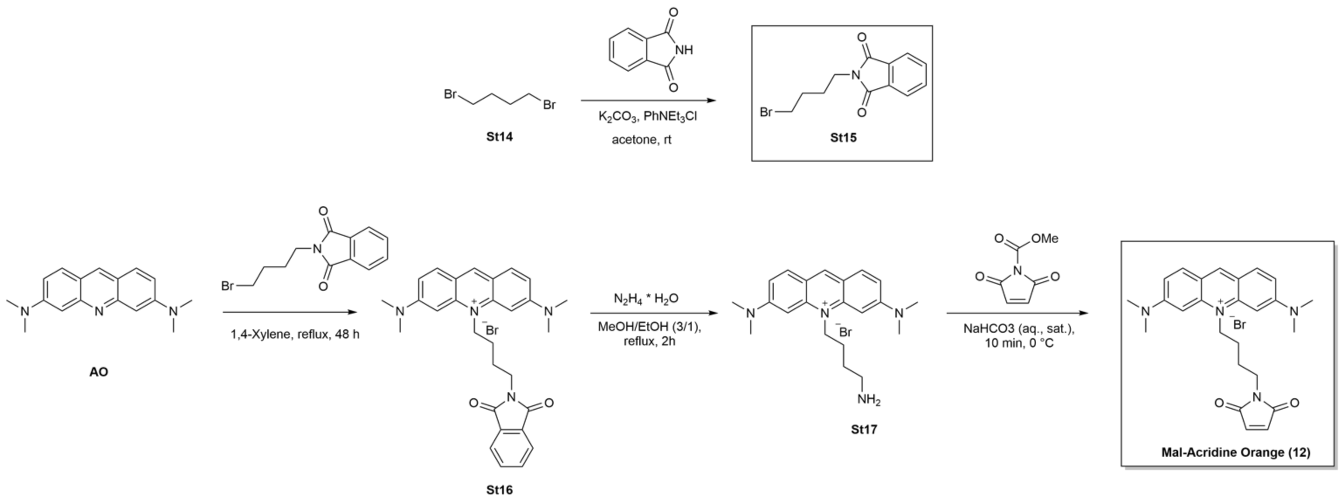

Figure S2: Synthesis of Mal-Acridine Orange.

Preparation of St15: Phthalimide (3000 mg, 20.4 mmol), K_2CO_3 (8450 mg, 61.2 mmol) and phenyltriethylammonium chloride (464 mg, 2.04 mmol) were suspended in acetone (100 ml). 1,4-Dibromobutane (7.2 ml, 61.2 mmol) was added to the suspension dropwise. The resulting mixture was stirred for 18 h at room temperature. After this time the solvent was removed under reduced pressure. The residue was taken up in water and CH_2Cl_2. The aqueous phase was extracted with CH_2Cl_2, the combined organic phases were dried over Na_2SO_4, filtered and the solvent was removed under reduced pressure. The crude material was purified over silica (PE/EA = 2/1). St15 (5008 mg, 17.8 mmol, 87 %) was isolated as a white solid.

^{1}H NMR (400 MHz, $CDCl_3$): δ 7.86 (dd, *J* = 5.4 Hz, 3.1 Hz, 2H), 7.74 (dd, *J* = 5.5 Hz, 3.0 Hz, 2H), 3.74 (t, *J* = 6.6 Hz, 2H), 3.46 (t, *J* = 6.3 Hz, 2H), 1.99 – 1.77 (m, 4H). The analytic data correspond to reporting literature.[8]

Preparation of St16: AO (Acridine Orange) (555 mg, 2.1 mmol) and St15 (1520 mg, 7.0 mmol) were dissolved in 1,4-Xylene (17 ml) and the solution was stirred under reflux conditions for 48 h. After completion, the resulting suspension was filtered over celite and the precipitate was

washed with toluene until the filtrate appeared colorless. The precipitate was washed off the celite with CH_2Cl_2/CH_3OH (1/1) and after removal of the solvent, St16 (1028 mg, 1.9 mmol, 90 %) was obtained as a red solid and was used without further purification. **^{1}H NMR (400 MHz, DMSO-*d6*):** δ = 8.76 (s, 1H), 7.90 (d, *J* = 9.3 Hz, 1H), 7.84 (t, *J* = 1.2 Hz, 4H), 7.23 (dd, *J* = 9.3 Hz, 2.0 Hz, 2H), 6.60 (s, 1H), 4.74 (t, *J* = 7.6 Hz, 2H), 3.68 (t, *J* = 7,2 Hz, 2H), 1.90 (m, 4H). **^{13}C NMR (400 MHz, DMSO-*d6*): δ** 168.6, 155.8, 143.3, 142.6, 134.9, 133.4, 131.9, 123.57, 116.9, 114.8, 93.0, 46.7, 25.9, 23.3. **HRMS (ESI):** *m*/*z* calculated for $C_{29}H_{31}N_4O_2^+$ = 467.2442; found: 467.2450

Preparation of St17: St16 (600 mg, 1.1 mmol) was dissolved in CH_3OH/CH_3CH_2OH (3/1, 11 ml). Hydrazine monohydrate (664 µl, 8.8 mmol was added to the solution and the mixture was stirred at 80 °C for 6 h. After this time, the solvents were removed under reduced pressure and the crude material was via reverse phase chromatography (C18, 35min, 0 – 45 % CH_3CN (+ 0,05 % TFA) in H_2O (+ 0,05 % TFA)). St17 (458 mg, quant.) was isolated as a red solid. **^{1}H NMR (400 MHz, DMSO-*d6*):** δ = 8.81 (s, 1H), 7.95 (s, 2H), 7.29 (dd, *J* = 9.3 Hz, 2.0 Hz, 2H), 6.67 (s, 2H), 5.72 (s, 6H), 4.79 (t, *J* = 7.6 Hz, 2H), 3.30 (s, 12H), 2.92 (q, *J* = 6.6 Hz, 2H), 1.95 (q, *J* =7.9 Hz, 2H), 1.83 (q, *J* = 8.0 Hz, 2H). **^{13}C NMR (101 MHz, DMSO-*d6*): δ** = 155.9, 143.4, 142.7, 133.5, 117.0, 114.8, 93.2, 46.4, 25.1, 23.5. **HRMS (ESI):** *m*/*z* calculated for $C_{21}H_{29}N_4^+$ = 337.2387; found: 337.2388.

Preparation of Mal-Acridine Orange (12): St17 (360 mg, 0.9 mmol) was dissolved in aq. $NaHCO_3$ (sat., 4.5 ml) at 0 °C. *N*-Methoxycarbonylmalimid (260 mg, 1.1 mmol) was added to the solution and the reaction was stirred for 10 min. The reaction mixture was lyophilized and the residue was loaded to celite and Mal-Acridine Orange was washed off with CH_2Cl_2/*i*PrOH (5/1 + 1 % TEA). The solvents were removed under reduced pressure and (12) was obtained as a red solid and was used without further purification. **^{1}H NMR (400 MHz, DMSO-*d*$_6$) δ** = 8.82 (s, 1H),

7.95 (d, J = 9.3 Hz, 1H), 7.29 (dd, J = 9.4, 2.0 Hz, 2H), 7.02 (s, 1H), 6.64 (s, 2H), 4.75 (s, 2H), 3.53 (t, J = 5.6 Hz, 2H), 3.28 (s, 12H), 1.97 – 1.68 (m, 4H). **^{13}C NMR (400 MHz, DMSO- d_6) δ** = 171.7, 168. 1, 165.7, 155.9, 142.6, 135.0, 117.0, 114.9, 93.0, 52.0, 49.1, 46.0, 26.1, 23.4. **HRMS (ESI):** m/z calculated for $C_{25}H_{29}N_4O_2^+$ = 417.2285; found: 417.2293

Analytical methods

Detection of Hoechst-PEG-Azide/ Mal-Acridine Orange on nanoparticles. To detect and quantify immobilized Hoechst-PEG-Azide/Mal-Acridine Orange on the particle surface, particles were dissolved in 90 v%/10 v% 1M NaOH/DMSO. To subtract any signal from unmodified nanoparticles, 1,5-Bistri(isopropoxysilyl)-benzene-3-thiol-alkyne particles were also dissolved in 90 v%/10 v% 1M NaOH/DMSO with the same concentration. The curve of unfunctionalized particles was subtracted from the curve of the dye-functionalized nanoparticles. To quantify the amount of immobilized dye, calibration curves of Hoechst-PEG-Azide and Mal-Acridine Orange were also recorded in 90 v%/ 10 v% 1M NaOH/DMSO.

Ellman assay. The standard protocol of the Ellman assay was used to test the accessible thiols of various MOPs-SH samples: samples without functionalized surface, samples with alkynes on the surface and blocked pores and samples with alkyne on the surface and extracted (accessible) pores. 3 mg nanoparticles were dissolved in 10 mL of 20 µM 5, 5'-dithiobis-(2-nitrobenzoic acid) solution (in 0.1 M phosphate buffer pH= 8). After stirring for one hour at room temperature, the suspension was centrifuged and the supernatant was analyzed by UV-Vis measurement.

Reactive oxygen species (ROS) detection via uric acid degradation. 3 mg of 1,5-Bistri(isopropoxysilyl)-benzene-3-thiol-alkyne-Hoechst-Acridine orange were suspended in 1.5 mL of a 1 mM uric acid solution. The suspension was irradiated for a defined period of time (2, 4,

6, 8, 10, 12, 14, 16, 18, 20, 25, 30, 35, 40, 45 min) with a blue LED. The suspension was centrifuged and the concentration of uric acid in the supernatant analyzed by UV-Vis measurement.

Singlet oxygen ($^1O_2$) detection via 9,10-anthracenediyl-bis(methylene)dimalonic acid (ABDA) degradation. 3 mg of 1,5-Bistri(isopropoxysilyl)-benzene-3-thiol-alkyne-Hoechst-Acridine orange were suspended in 1.5 mL of a 165.7 µM ABDA (in PBS, pH = 7.4) solution. The suspension was irradiated for a defined period of time (0, 5, 10, 15, 20, 25, 30, 35, 40, 45, 50, 55, 60, 70 min) with a blue LED. The suspension was centrifuged and the concentration of ABDA in the supernatant analyzed by UV-Vis measurement.

Biofilm Growth. Depending on the purpose of the experiment, two different methods to grow a biofilm were performed. Both are described below.

Biofilm imaging: A preculture of *Pseudomonas Fluorescens* WCS35 was grown by inoculating ~ 10 ml of LB medium from a cryostock and subsequent incubation of the preculture in an orbital shaker (180 r.p.m.) at 37 °C overnight (~16 h). After this time, the overnight culture was diluted with fresh LB medium to an OD_{600} of 0.075.

An autoclaved glass cover slip (10 x 10 mm) was placed in a 6 well plate and the cover slip was overlaid with 2 ml of the diluted cell culture. The plate was placed in an incubator and was allowed to incubate for 24 h. After this time, particles (c = 50 µg/ml) were added and incubated for further 30 min at room temperature. The cover slip was salvaged from the well and rinsed with water to remove unbound particles and bacteria.

As a negative control, an uncultured coverslip was overlaid with 2 ml of LB medium and treated as described above. The imaging was performed with a Cytation 5-plate Reader (Biotek) using a brightfield lamp and a DAPI filter cube (ex/em: 377/50 nm / 447/60 nm).

Incubation/Irradiation of a biofilm: A preculture of *Pseudomonas Fluorescens* WCS35 was grown by inoculating ~ 10 ml of LB medium from a cryostock and incubating the preculture in an orbital shaker (180 r.p.m.) at 37 °C overnight (~16 h). After this time, the overnight culture was diluted with fresh LB medium to an OD_{600} of 0.075. A 96 well plate was inoculated with 200 µl of the diluted preculture. The plate was placed in an incubator and was allowed to incubate for 24 h. After this time, particles (c = 0, 10, 50, 100 and 250 µg/ml) were added and incubated for further 30 min at room temperature. An untreated and unirradiated biofilm, as well as an unirradiated but incubated (c = 250 µg/ml) biofilm were used as references. Every determination was performed in triplicates of biologically independent samples.

The supernatant was removed and the residue was rinsed with water once, to remove unbound particles and bacteria. The plate was then placed under a LED construction (comprised of 6 LEDs) and was irradiated with blue LED (470 mW, for spectrum see **Figure S3**) for 180 min (see **Figure S4).**

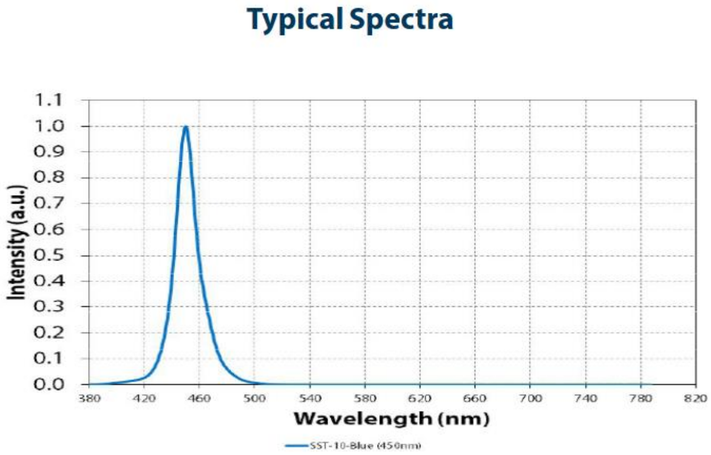

Figure S3. Spectrum for LED that was used in irradiation experiments.

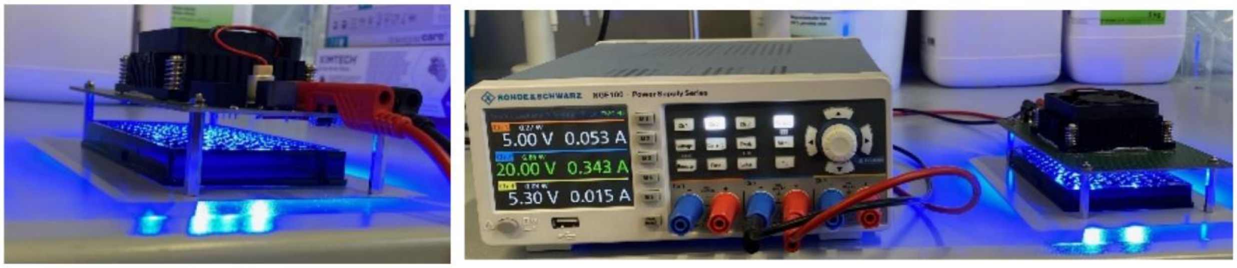

Figure S4. 2 LED construction with 96 well plate beneath.

To determine the number of living bacteria after the treatment, a CFU counting was performed. For this purpose, 200 µl of fresh LB media was given in each well and was pipetted vigorously up and down for 30 seconds to resuspend the residual biofilm.

The biofilms were diluted 1:8000 for the references and the irradiated biofilms for the concentrations 0 – 50 µg/ml. The biofilms incubated with the concentrations 100 and 250 µg/ml were diluted 1:1000. Of each well aliquots of 50 µl were streaked out on agar plates and the plates were allowed to incubate at 30 °C for 24 h. The number of formed colonies were counted. The mean and the standard deviation are presented.

CHO-cells growth. 20 ml of culture medium (89 vol.% Ham's F-12 nutrition mix (Gibco), 10 vol.% FBS (Gibco), 1 vol.% Gentamicin (c = 50 µg/ml; Sigma Aldrich)) in a T75 culture bottle, were inoculated with 10^6 CHO-cells from a cryostock and were incubated at 37 °C in a 5 % CO_2 atmosphere for 3 d, during which time the cells became adherent. After this time, the residual medium was removed and the cells were rinsed with PBS once. To detach the cells, the growth surface of the bottle was covered with an Accutase solution (5 ml, 400 – 600 units/ml, Sigma Aldrich) and incubated for 5 min. 5 ml of fresh culture medium was added for deactivation of the protein. The suspension was centrifuged and the supernatant was removed. The pallet was resuspended in fresh medium and from this suspension, a 96-well plate was inoculated with 10^4 cells in 200 µl of medium. The plate was incubated at 37 °C in a 5 % CO_2 atmosphere for 3 d, after which time the cells became adherent again. The supernatent was removed and the cells were rinsed with PBS.

Cell permeability assay. For these experiments two working solutions were prepared. a) A 1 mg/ml Hoechst33342 stock solution was diluted 1:200 (final concentration: c = 9,4 µM) with the culture medium and b) a 1 mg/ml suspension of particles in culture medium. The cells were prepared as described above. The wells were filled with 200 µl of solutions a) or b) and were incubated for 10 min at 37 °C in a 5 % CO_2 atmosphere. The incubation solutions were removed and the cells were rinsed with PBS once. The imaging was performed with a Cytation 5-plate Reader (Biotek) using a brightfield lamp and a DAPI filter cube (ex / em: 377/50 nm / 447/60 nm).

Fluorescence lifetime measurements. For the FLT measurements the samples were dispersed in water and filled in 500 µL quartz cuvettes (10 mm path length). An Edinburgh FLS 1000 equipped with a MCP-PMT detector was used to measure with an EPL 375 nm pulsed laser and a pulse period of 100 ns using time-correlated single photon counting (TCSPC). In addition to the

sample measurements the instrument response function (IRF) was measured at the excitation wavelength of the laser (375 nm) and subsequently used for a reconvolution fit of the sample decays using the Edinburgh Fluoracle software and the exponential model function (1),

$$S(t) = \int_0^t E(t')R(t-t')\,dt' \quad (1)$$

where $S(t)$ is the measured fluorescence decay, $E(t)$ is the measured instrumental response function (IRF) and $R(t)$ is the theoretical sample decay model function, which is a tri exponential function (2).

$$R(t) = A_1 \cdot e^{-\frac{t}{\tau_1}} + A_2 \cdot e^{-\frac{t}{\tau_2}} + A_3 \cdot e^{-\frac{t}{\tau_3}} \quad (2)$$

The extracted fit parameters were extracted and are shown in table S1. The fluorescent lifetimes τ and amplitudes A were used to calculate the amplitude weighted lifetime $< \tau >$ and the average lifetime $\bar{\tau}$ with equation (3) and (4), respectively.

$$< \tau >= A_1\tau_1 + A_2\tau_2 + A_3\tau_3 \quad (3)$$

$$\bar{\tau} = \frac{A_1{\tau_1}^2 + A_2{\tau_2}^2 + A_3{\tau_3}^2}{A_1\tau_1 + A_2\tau_2 + A_3\tau_3} \quad (4)$$

The amplitude weighted lifetimes were then used for the calculation of the FRET efficiencies E_{FRET} with equation (5),

$$E_{FRET} = 1 - \frac{< \tau >_{DA}}{< \tau >_{D}} \quad (5)$$

where $<\tau>_{DA}$ and $<\tau>_{D}$ are the amplitude weighted lifetimes of the donor (Hoechst) in the presence and absence of the acceptor (Acridine Orange) i.e. with and without energy transfer respectively. The received FRET efficiencies are shown in table S2.

Supporting Figures

Figure S5: Characterization of 1,5-Bistri(isopropoxysilyl)-benzene-3-thiol (1).

(a) ^{1}H-NMR (400 MHz, $CDCl_3$) spectrum of 1,5-Bistri(isopropoxysilyl)-benzene-3-thiol.

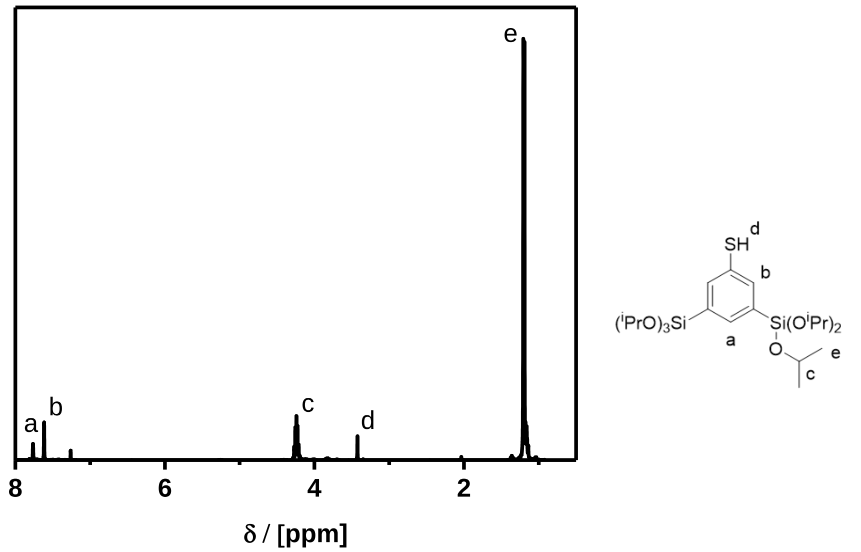

(b) ^{13}C NMR (400 MHz, $CDCl_3$) spectrum of 1,5-Bistri(isopropoxysilyl)-benzene-3-thiol.

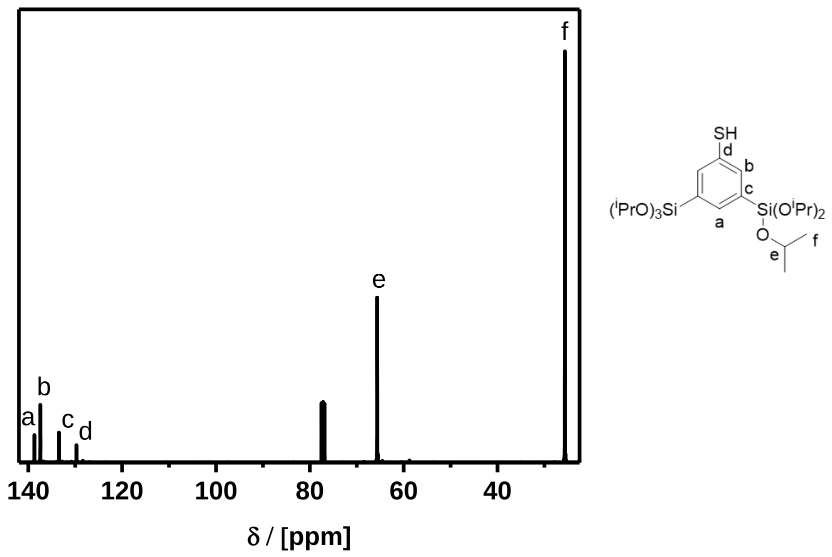

(c) ^{29}Si NMR (400 MHz, $CDCl_3$) spectrum of 1,5-Bistri(isopropoxysilyl)-benzene-3-thiol.

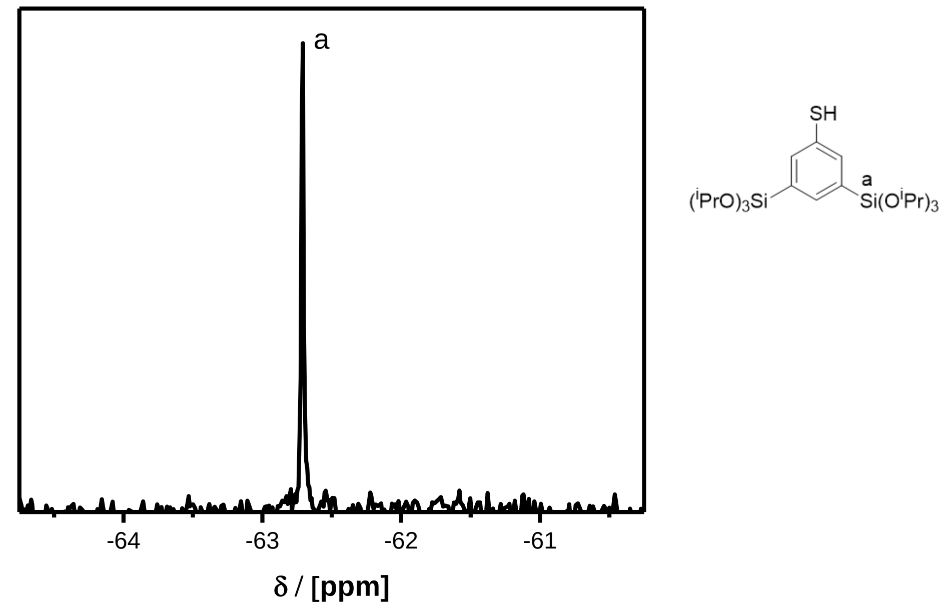

(d) ESI-MS spectrum of 1,5-Bistri(isopropoxysilyl)-benzene-3-thiol.

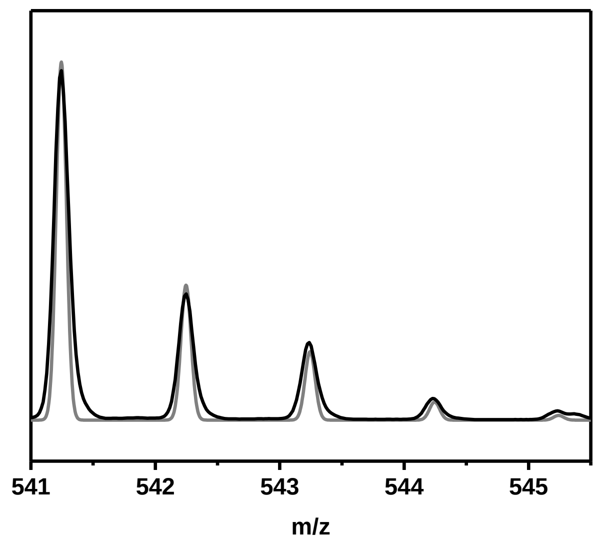

Black: measured spectrum
Grey: simulated spectrum

Figure S6. Characterization of MOPs-SH.

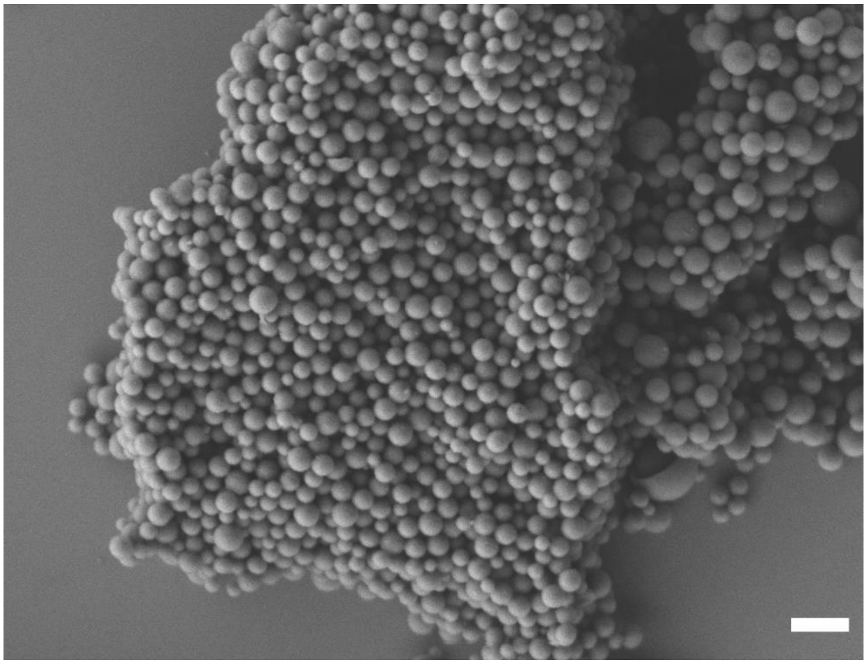

(a) SEM image; scale bar: 1 μm

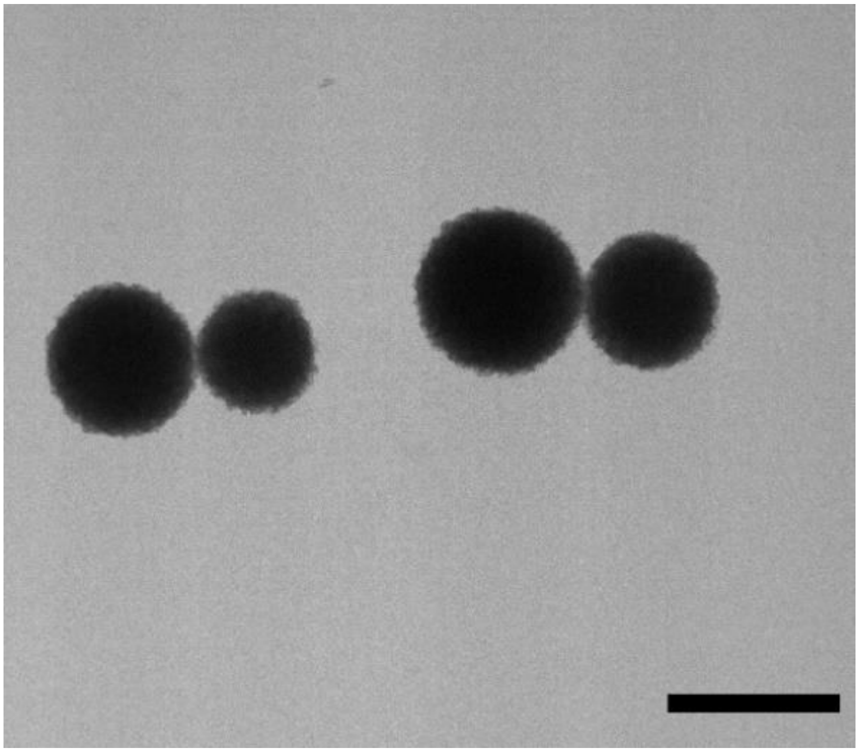

(b) TEM micrograph; scale bar: 500 nm

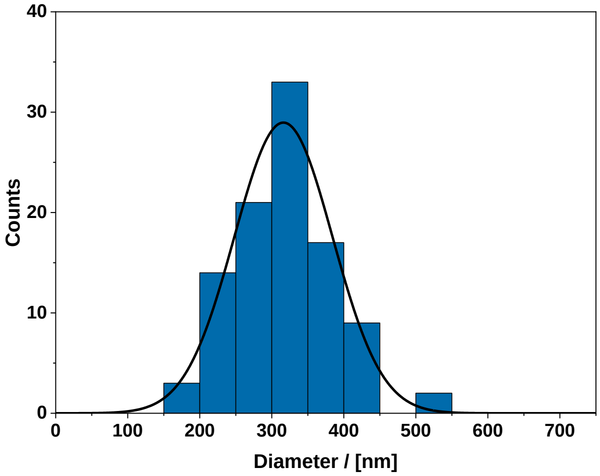

(c) Particle size distribution analysis from TEM data; counting of 100 particles; mean diameter = 316 nm; SD = 68 nm

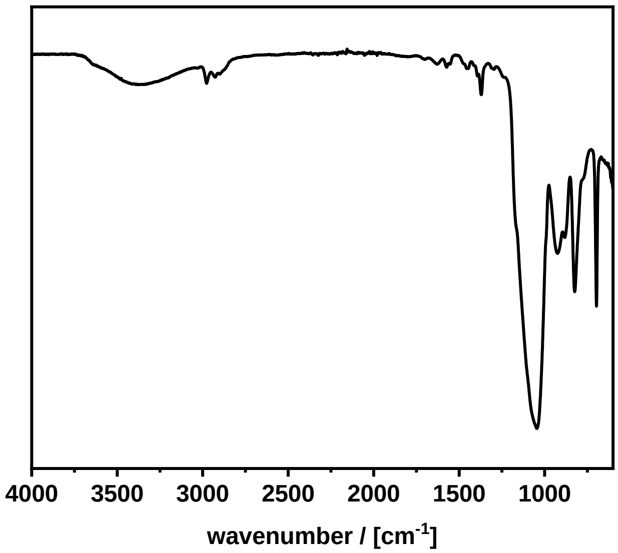

(d) ATR-IR spectrum; SiOSi vibration at ν = 1052 cm^{-1}; aliphatic CH vibrations at ν = 2986 - 2847 cm^{-1}; OH vibration at ν = 3300 cm^{-1}.

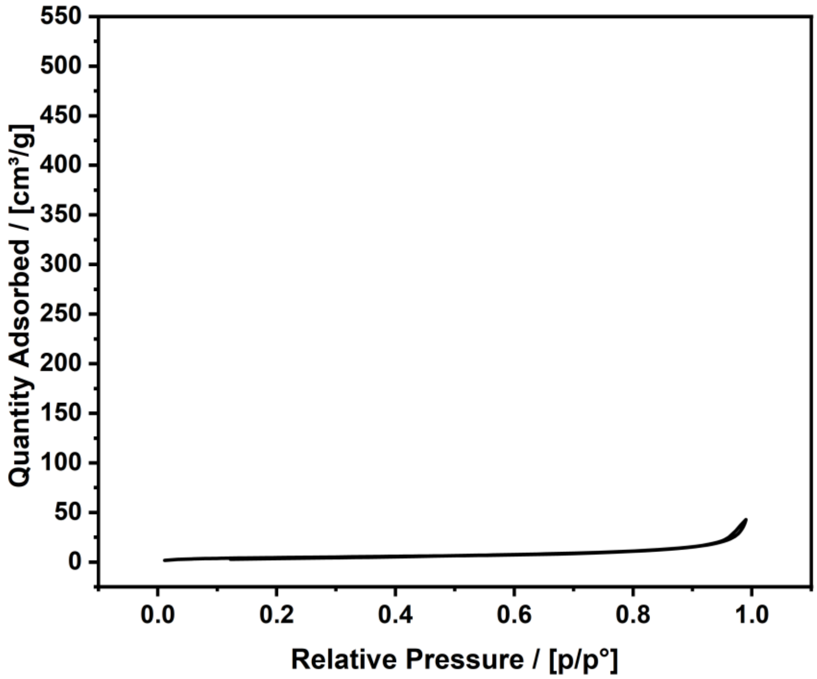

(e) N_2 physisorption isotherm of the material prior to template extraction.

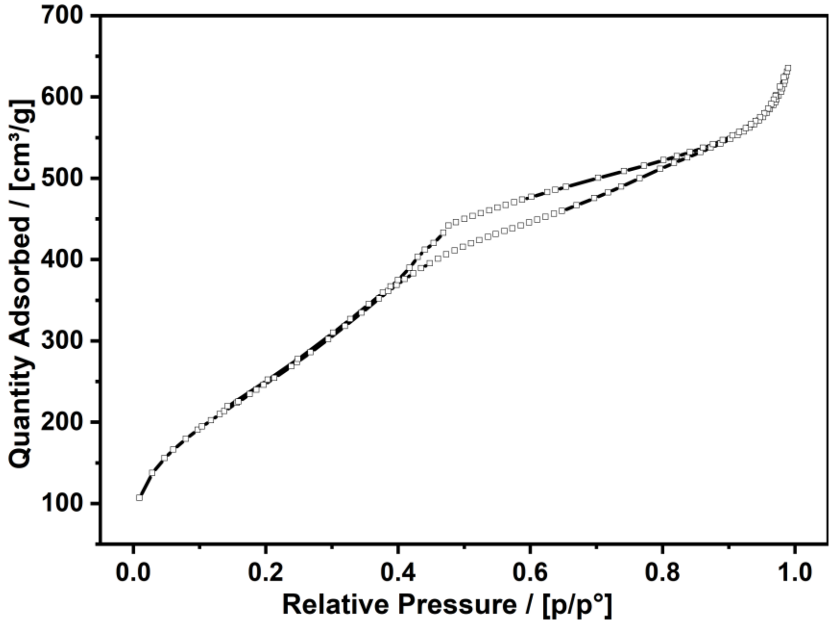

(f) N_2 physisorption isotherm of the material after template extraction.

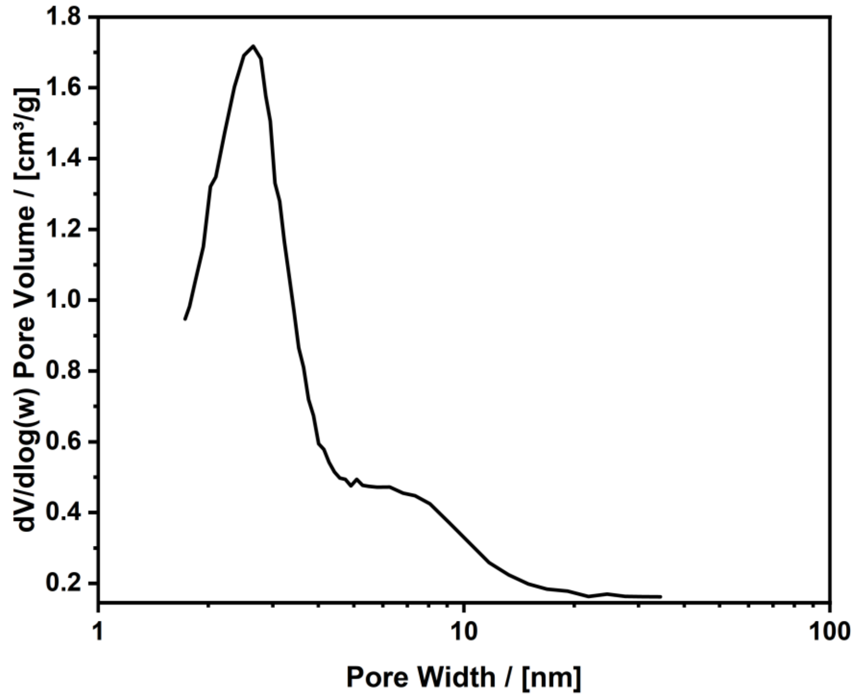

(g) Pore-size distribution function determined from N_2 physisorption isotherm

Figure S7. Characterization of *N*-Propargylmaleimide (2).

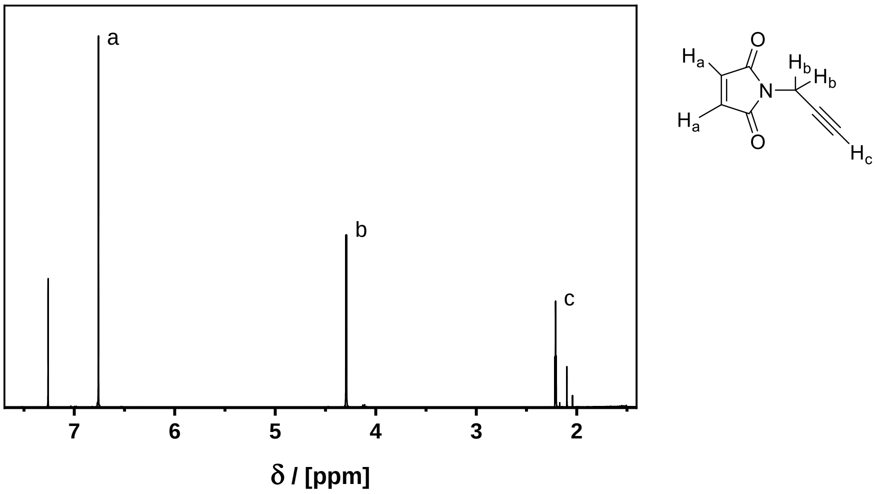

^{1}H NMR (400 MHz, $CDCl_3$) spectrum of N-Propargylmaleimide

Figure S8. Characterization of YN-MOPs-SH.

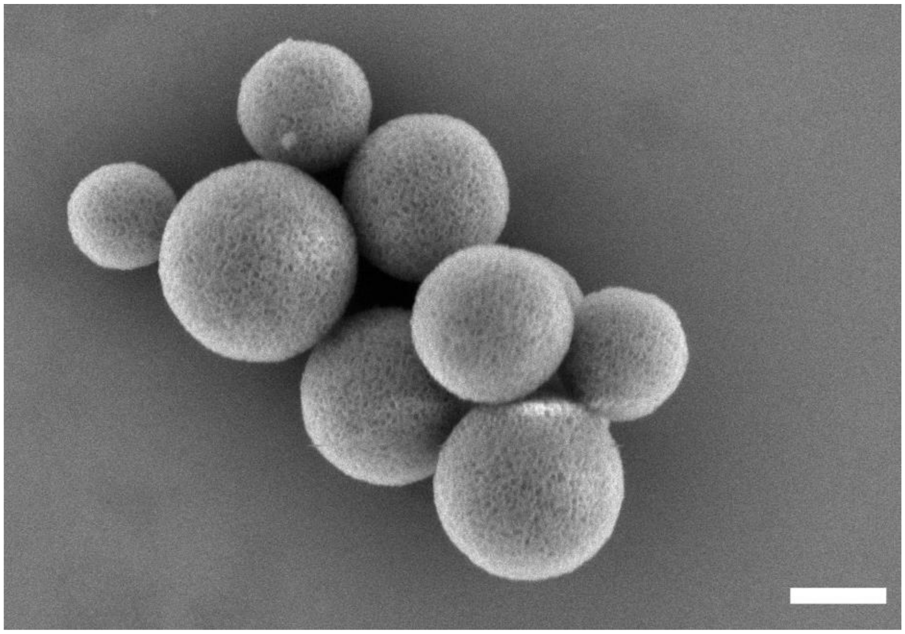

(a) SEM micrograph; Scale bar represents 200 nm.

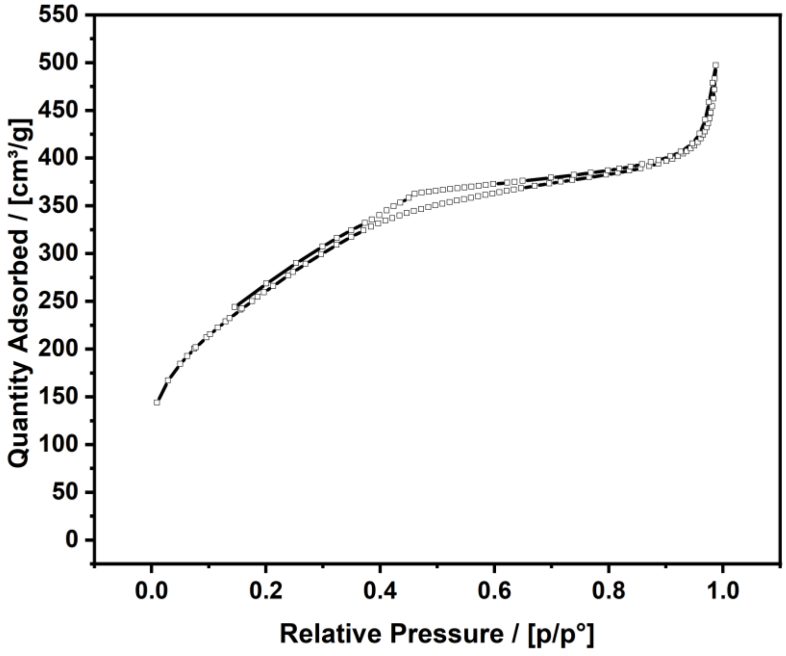

(b) N_2-physisorption measurements

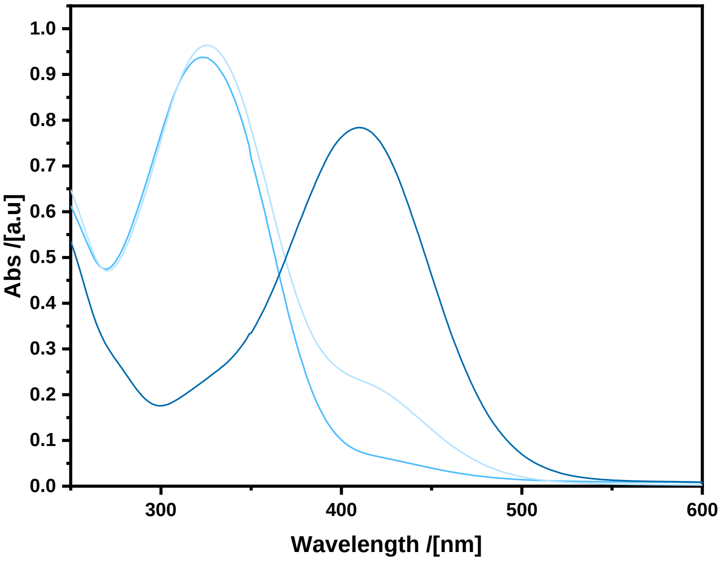

(c) Ellmann Essay. Dark blue: MOPs-SH nanoparticles filled with template (right after synthesis); Middle blue: YN-MOPs-SH nanoparticles (filled with template and alkyne on surface); Light blue: YN-MOPs-SH nanoparticles without template (free pores and alkyne on surface).

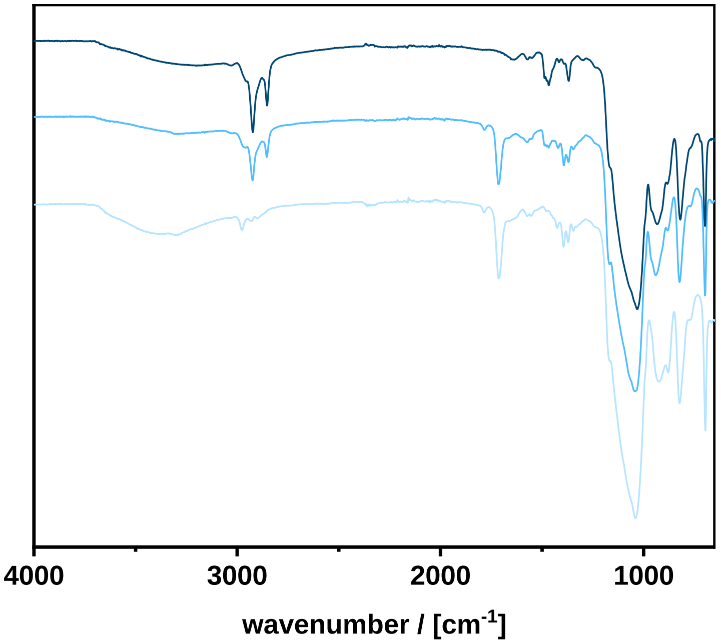

(d) AT-IR spectroscopy; dark blue: MOPs-SH nanoparticles filled with template (right after synthesis); middle blue: YN-MOPs-SH nanoparticles (filled with template and alkyne on surface); light blue: YN-MOPs-SH nanoparticles without template (free pores and alkyne on surface)

Sample	Wavenumber / [cm^{-1}]	vibration
Dark blue	2925	C-H (template)
	2850	C-H (template)
	1470	C-O (template)
	1040	Si-O (nanoparticle)
Middle blue (only additional band)	1715	C=O (maleimide)
Light blue	Disappearance of bands from template	

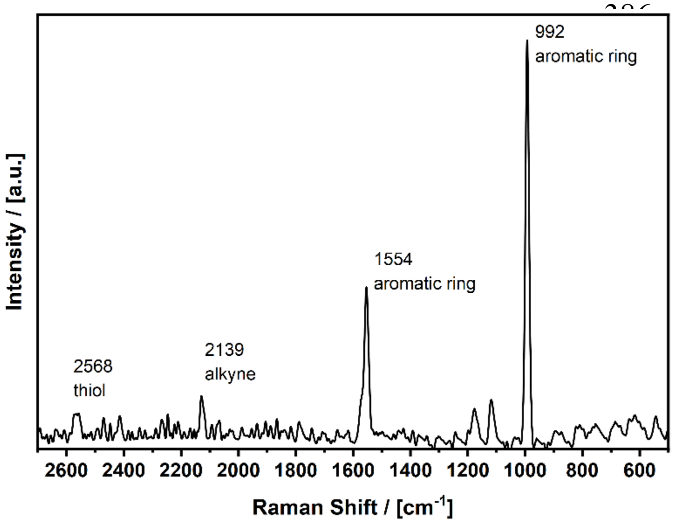

(e) Raman spectroscopy

Raman shift / [cm^{-1}]	vibration
2568	Thiol
2139	Alkyne
1554	Aromatic ring
992	Aromatic ring

Figure S9. Analytical data for the intermediates obtained at the different steps leading to Hoechst-PEG-Azide/ compound (**7**).

(a) ^1H NMR (400 MHz, $CDCl_3$) spectrum of St2

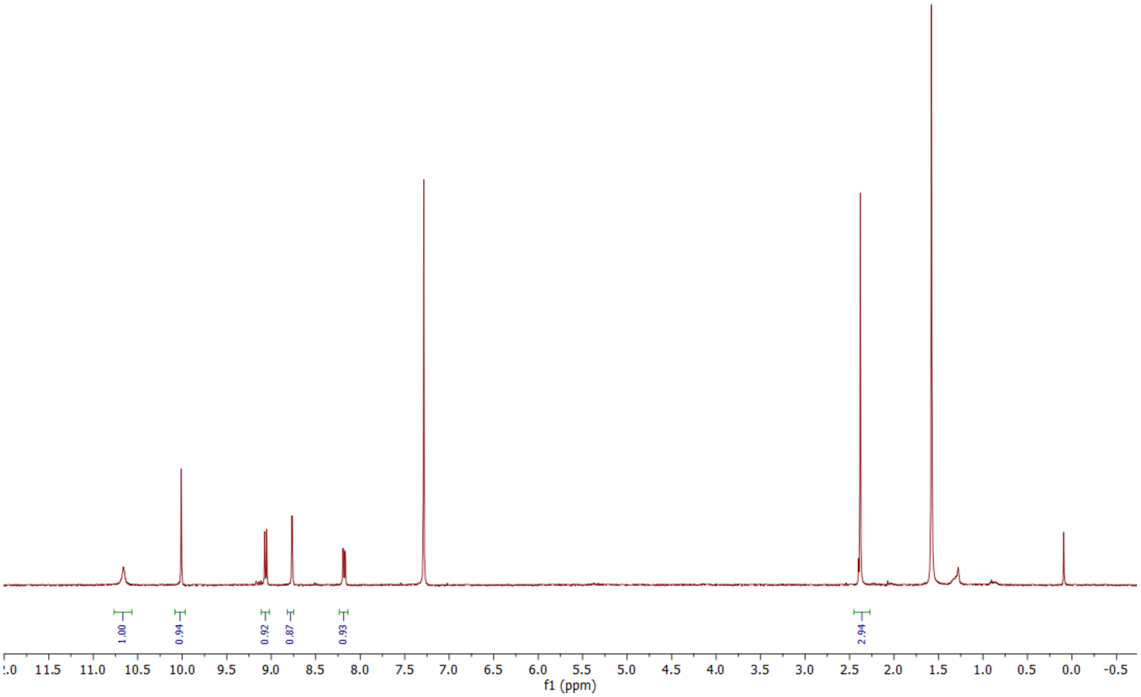

(b) ^{1}H NMR (400 MHz, DMSO-d_6) spectrum of St3

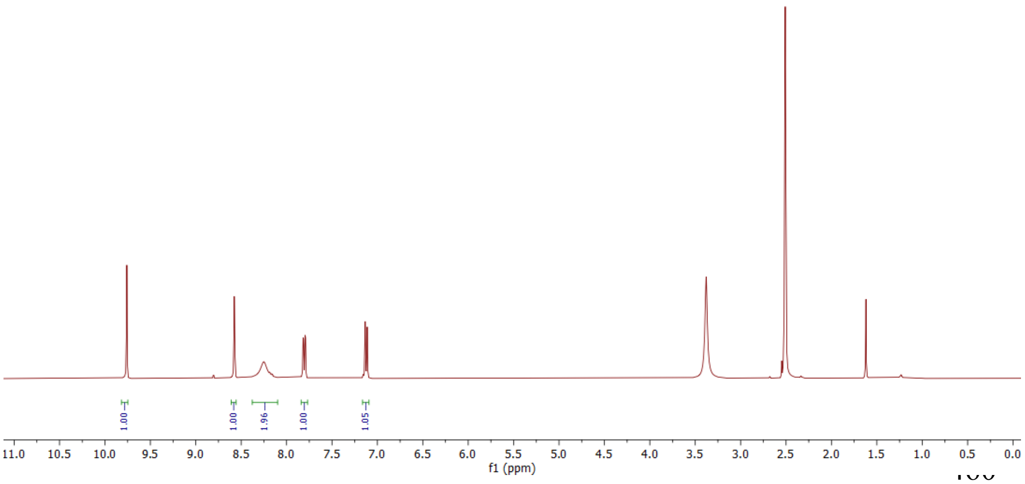

(c) ^{1}H NMR (400 MHz, $CDCl_3$) spectrum of St5

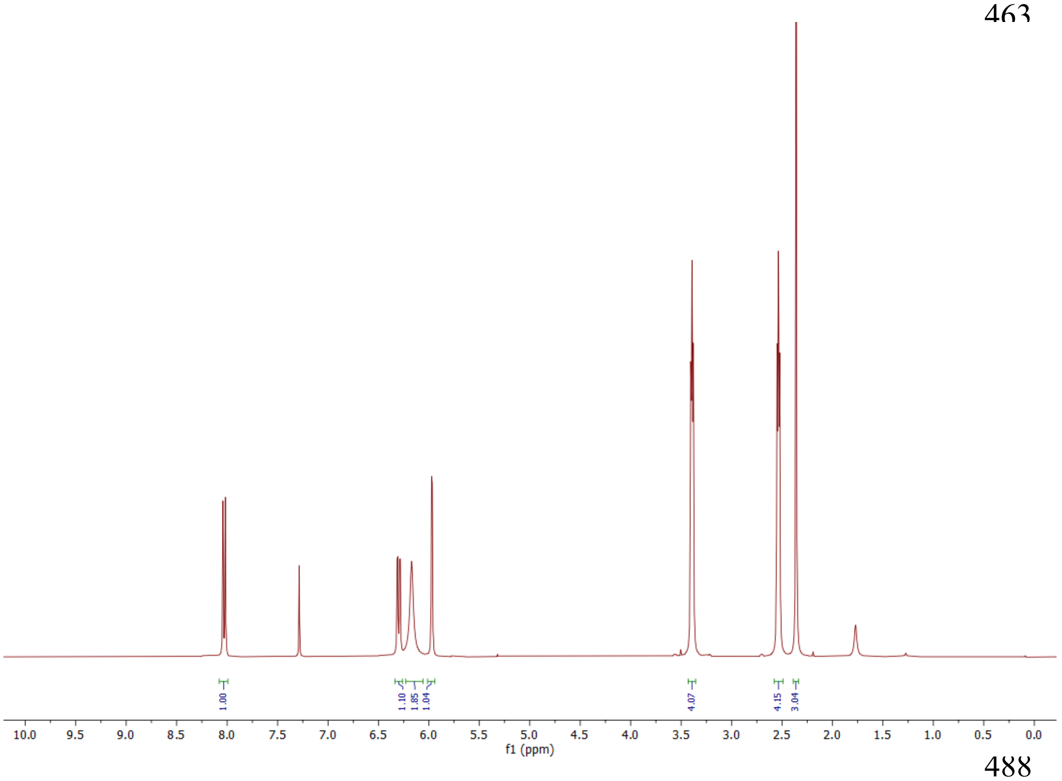

(d) ^{1}H NMR (400 MHz, $CDCl_3$) spectrum of St8

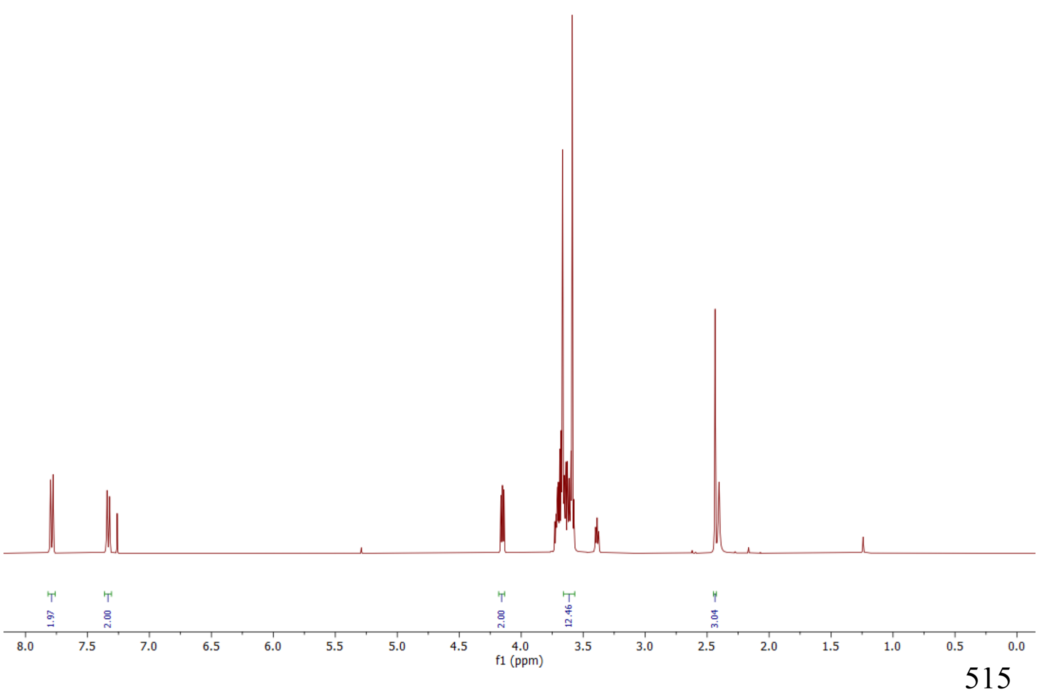

(e) ^{1}H NMR (400 MHz, $CDCl_3$) spectrum of St9

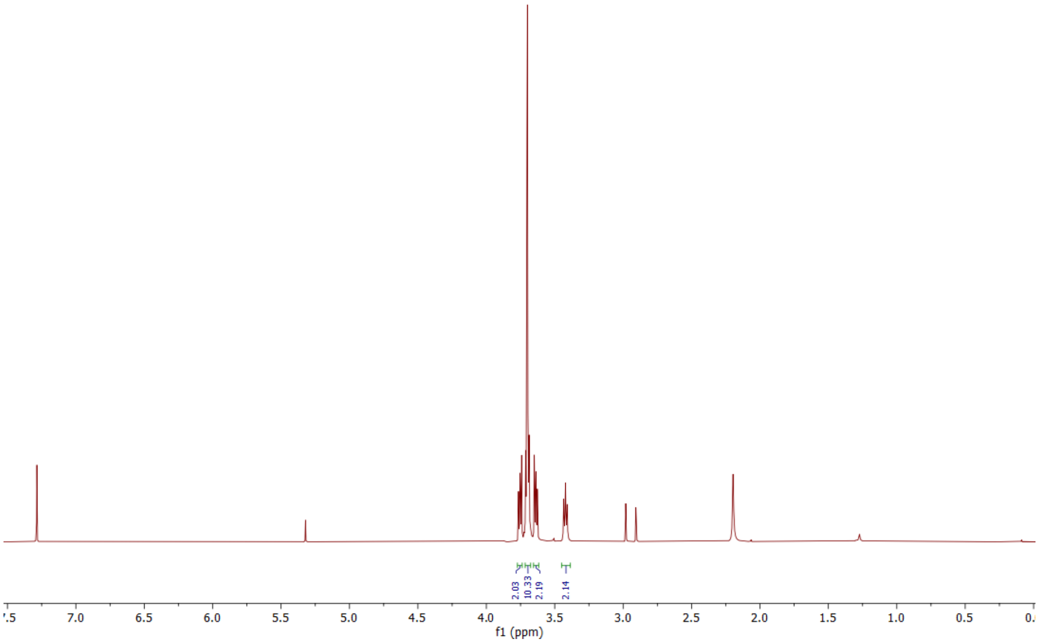

(f) ^{1}H NMR (400 MHz, $CDCl_3$) spectrum of St10

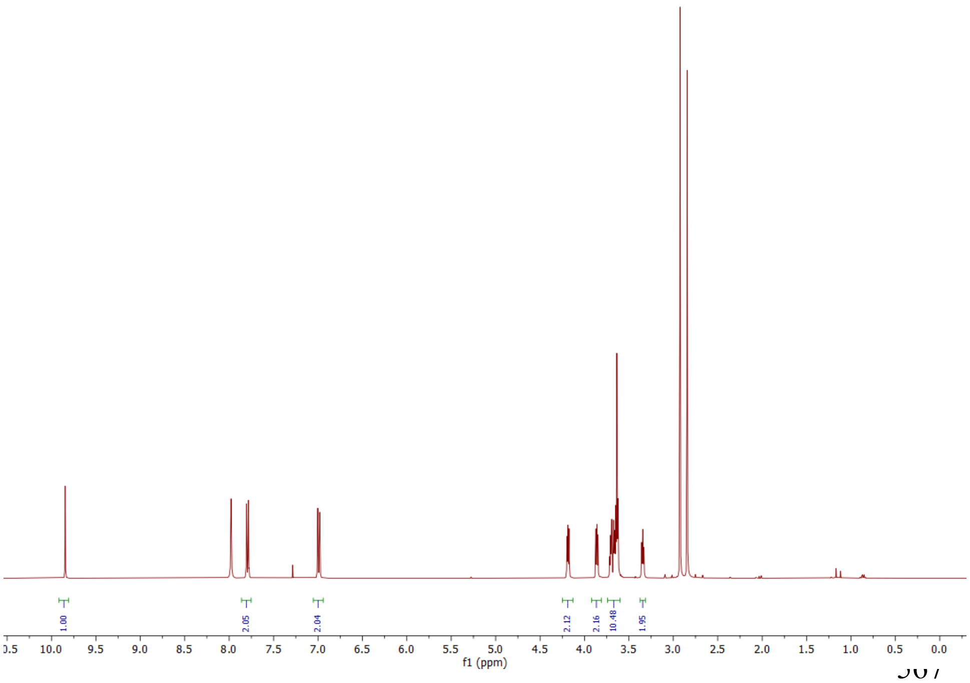

(g) ^{13}C NMR (400 MHz, CDCl3) spectrum of St10

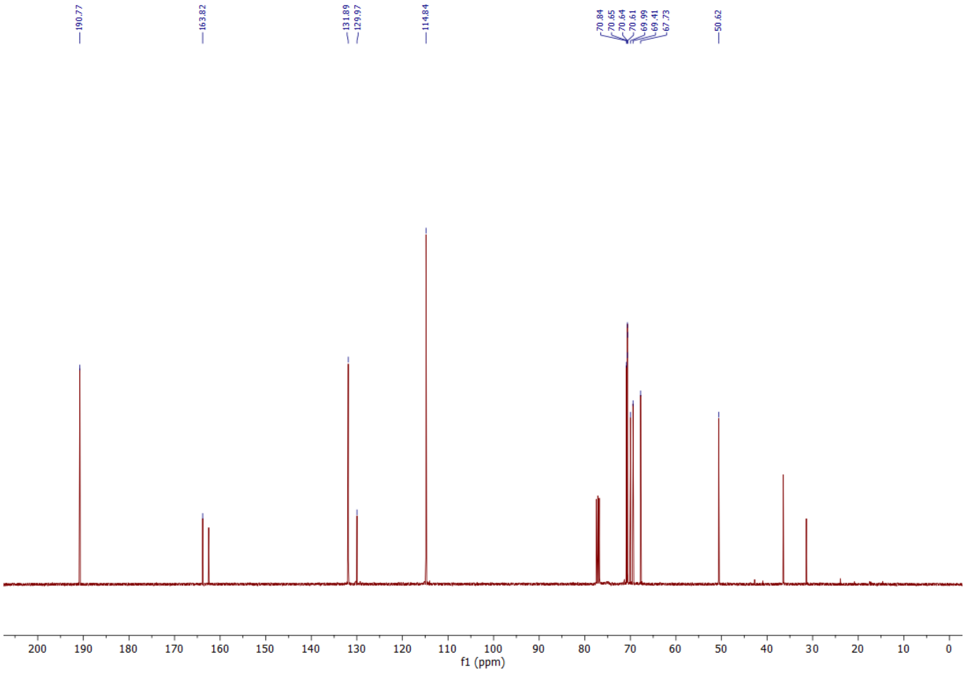

(h) ^{1}H NMR (400 MHz, DMSO-d_6) spectrum of St11

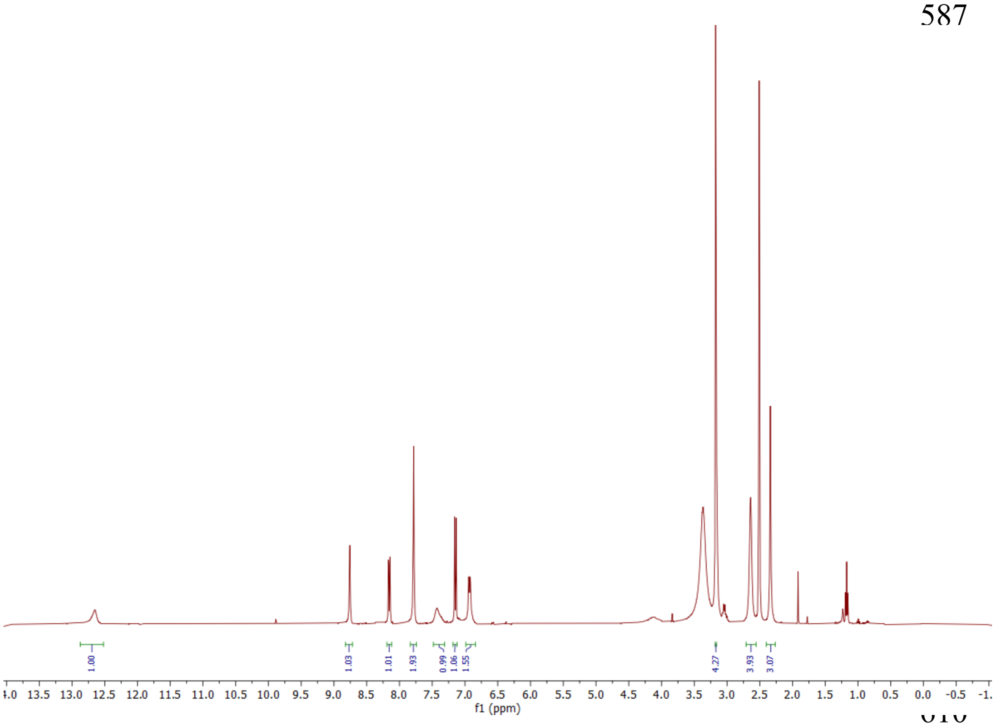

(i) ^{13}C NMR (400 MHz, DMSO-d_6) spectrum of St11

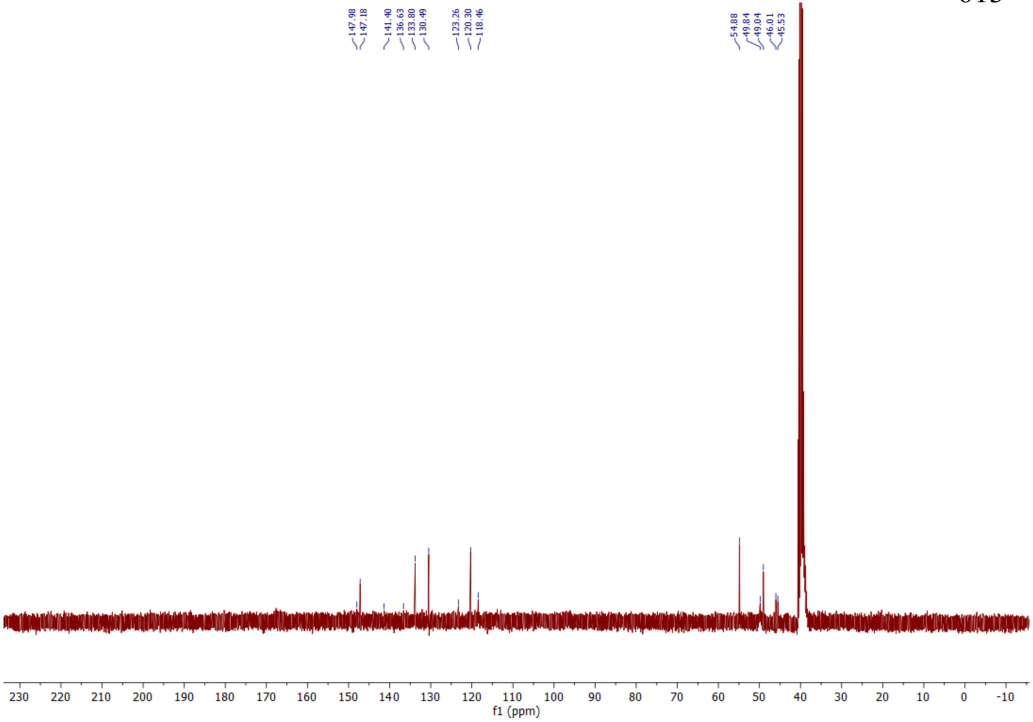

(j) ^{1}H NMR (400 MHz, MeOD) spectrum of Hoechst-PEG-Azide (7)

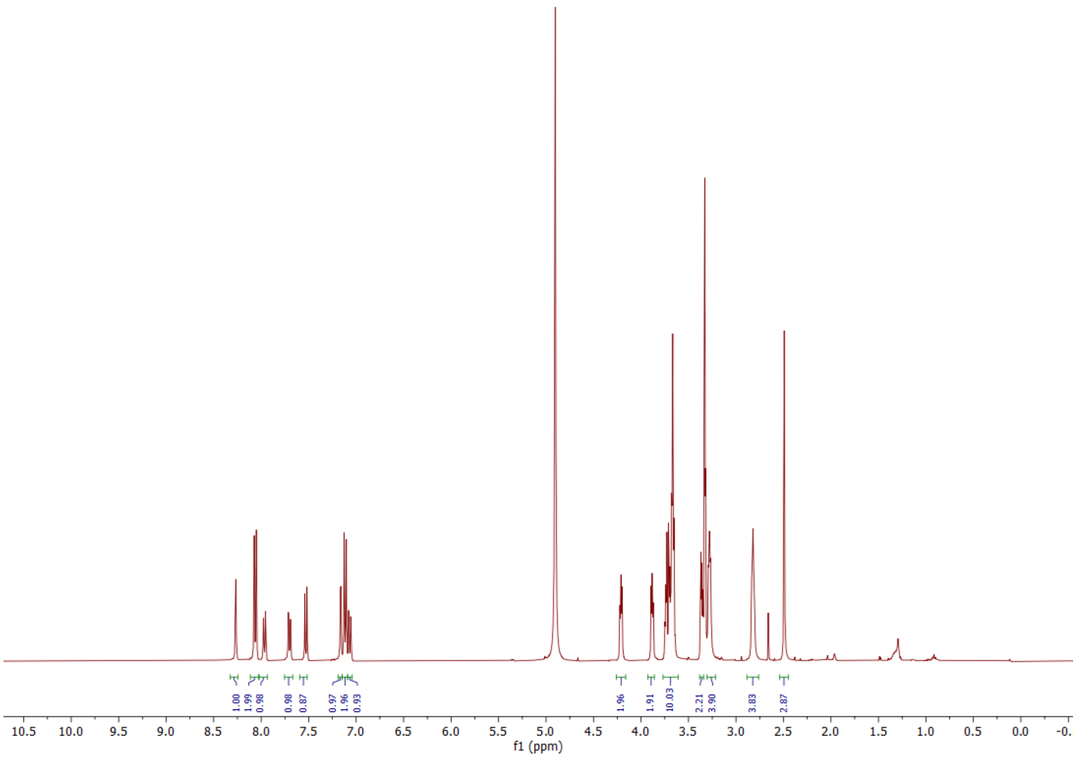

(k) ^{13}C NMR (400 MHz, MeOD) spectrum of Hoechst-PEG-Azide

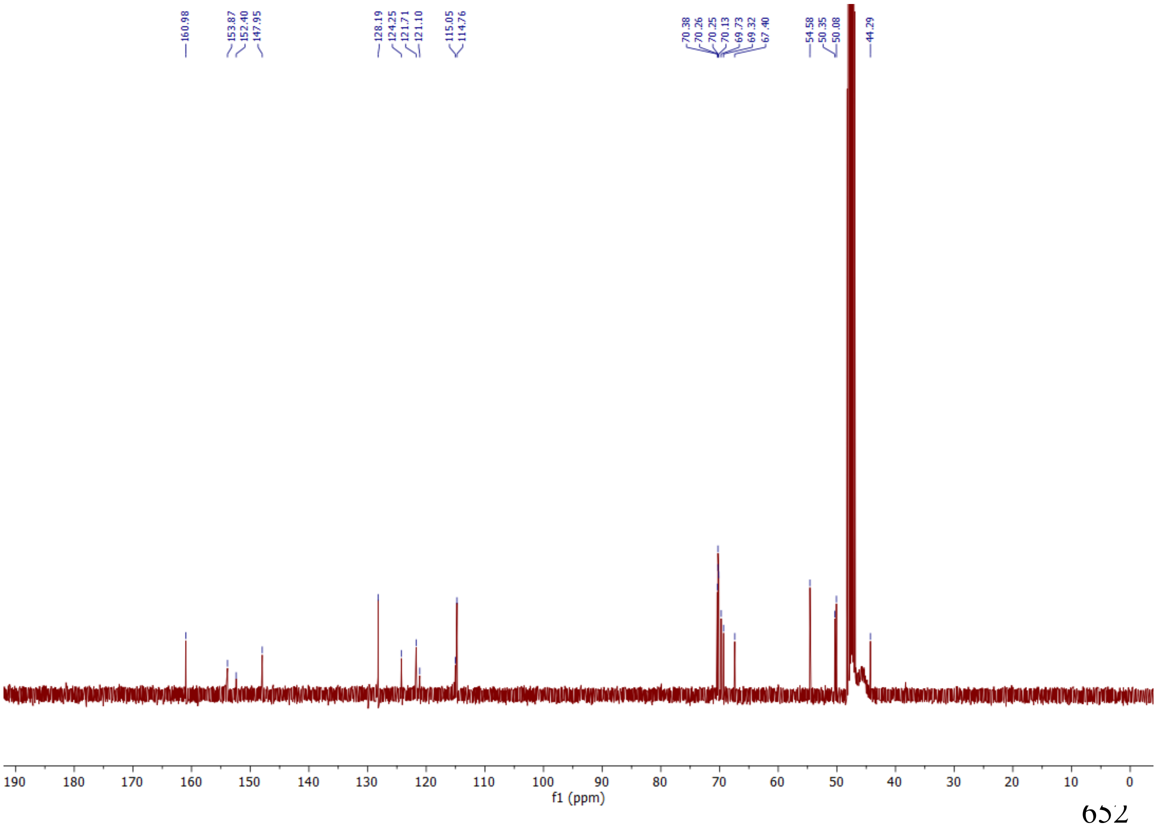

Figure S10. Characterization of HO-MOPs-SH.

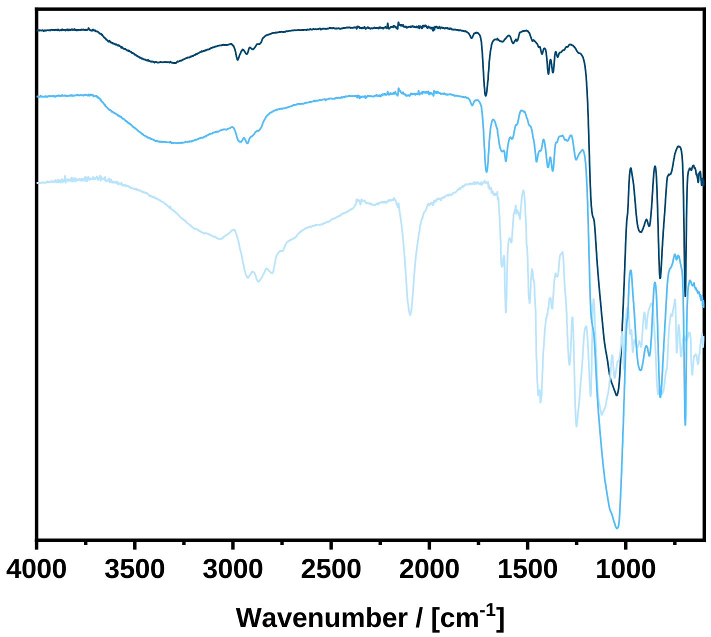

(a) ATR-IR

Dark blue: YN-MOPs-SH; Middle blue: HO-MOPs-SH; Light blue: Hoechst-PEG-Azide (**7**)

Assignment of bands:

Sample	Wavenumber / [cm^{-1}]	vibration
Dark blue	1030	Si-O (nanoparticle)
	1715	C=O (maleimide)
Middle blue (only	1455	Triazole[9]
additional bands)	1612	Triazole

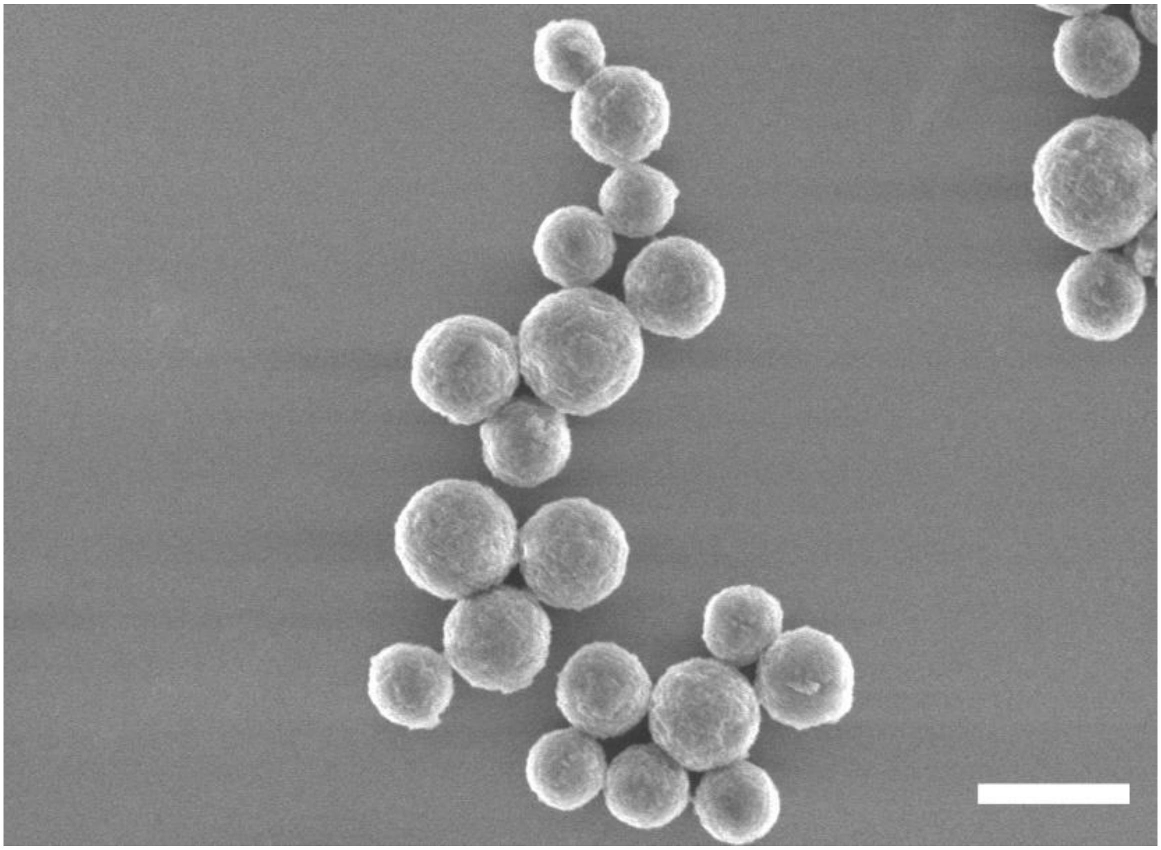

(b) SEM image of HO-MOPs-SH nanoparticles; Scale bar: 500 nm

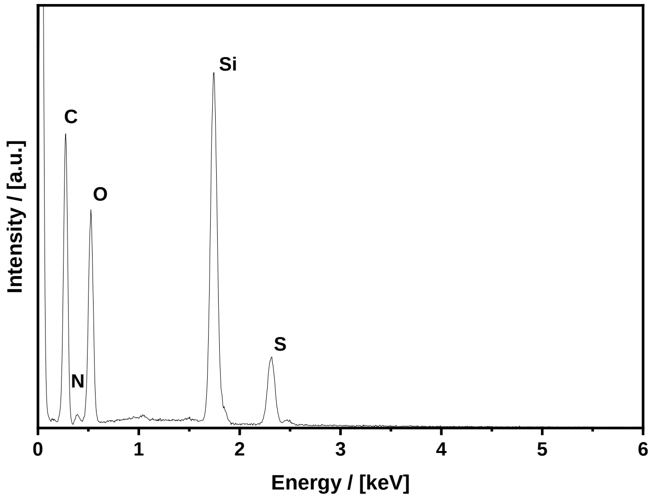

(c) EDX spectrum of HO-MOPs-SH nanoparticles confirming the absence of a Cu signal

Sulfur signal can be attributed to the thiol-containing nanoparticles, Nitrogen results from the amine in the maleimide group of the N-Propargylmaleimide and the amines of Hoechst-PEG-Azide.

Figure S11. Characterization of Mal-Acridine Orange.

(a) ^{1}H NMR (400 MHz, $CDCl_3$) spectrum of St15

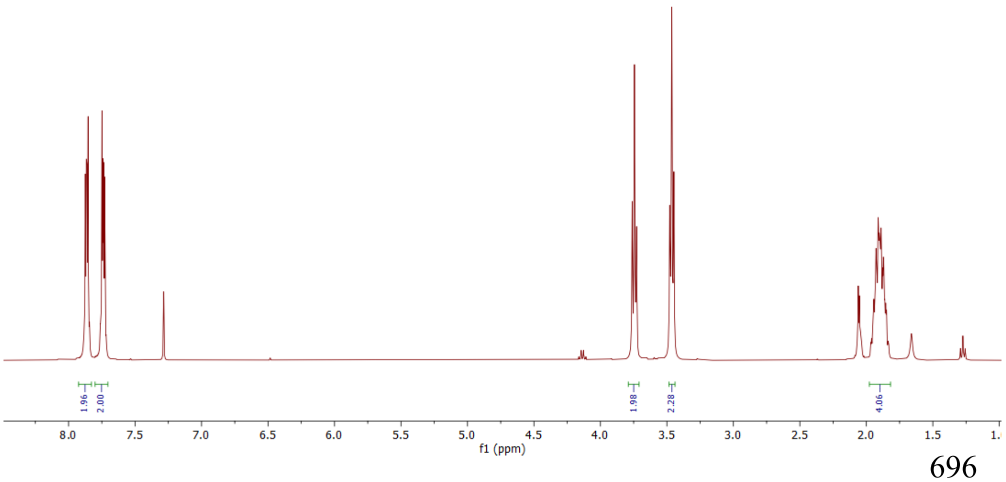

(b) ^{1}H NMR (400 MHz, DMSO-d_6) spectrum of St16

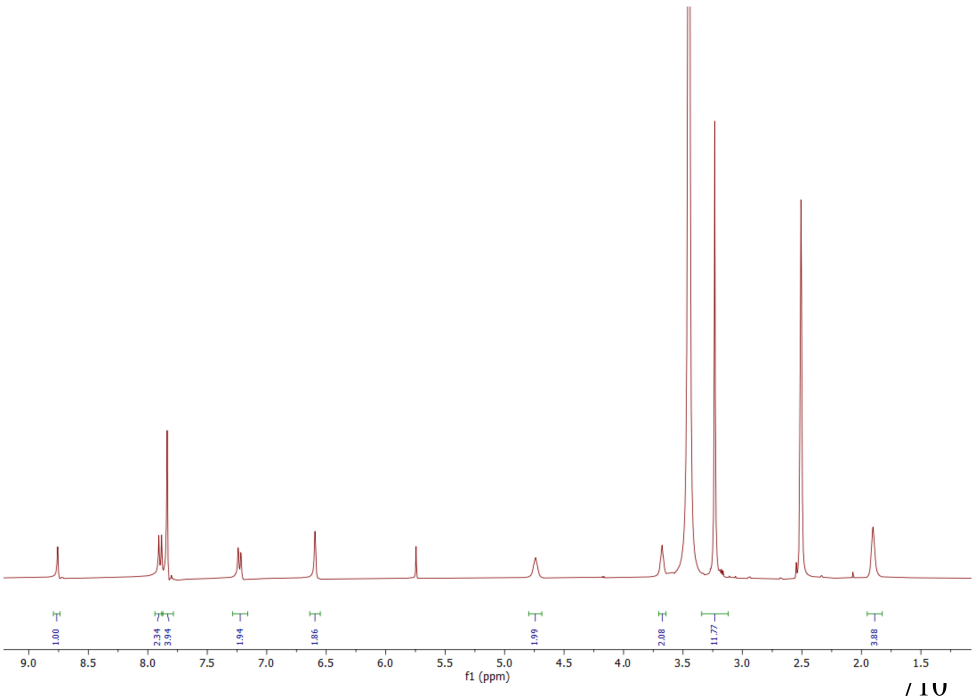

(c) ^{13}C NMR (400 MHz, DMSO-d_6) spectrum of St16

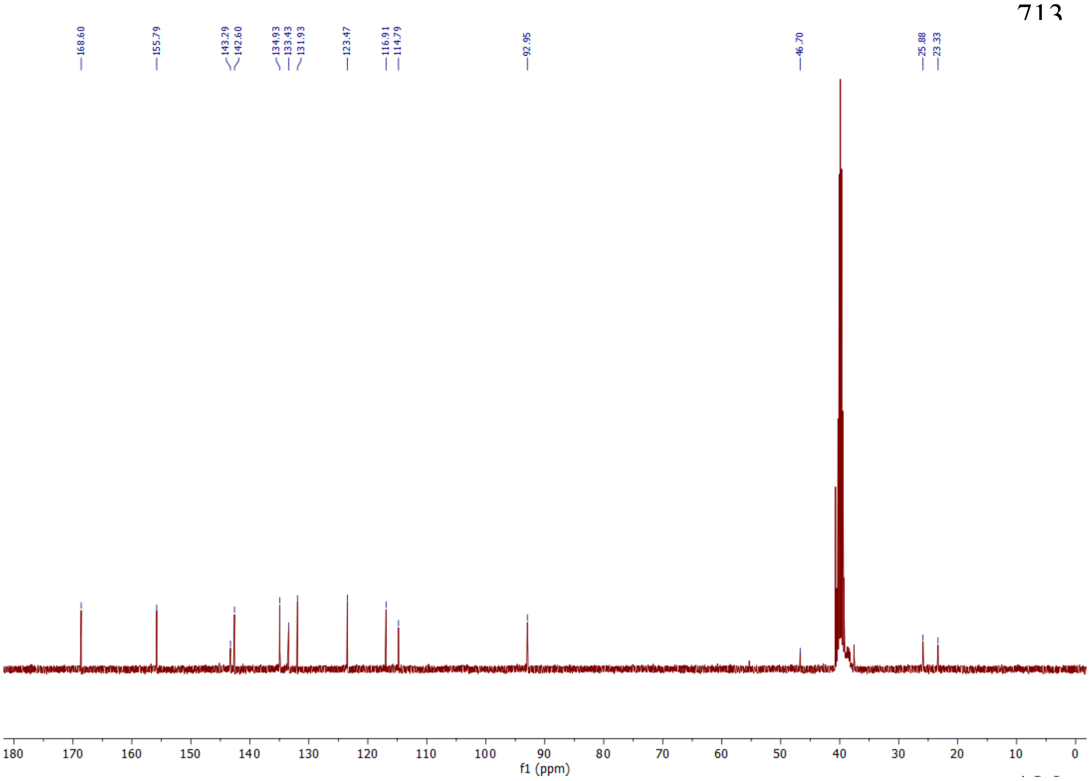

(d) ^{1}H NMR (400 MHz, DMSO-d_6) spectrum of St17

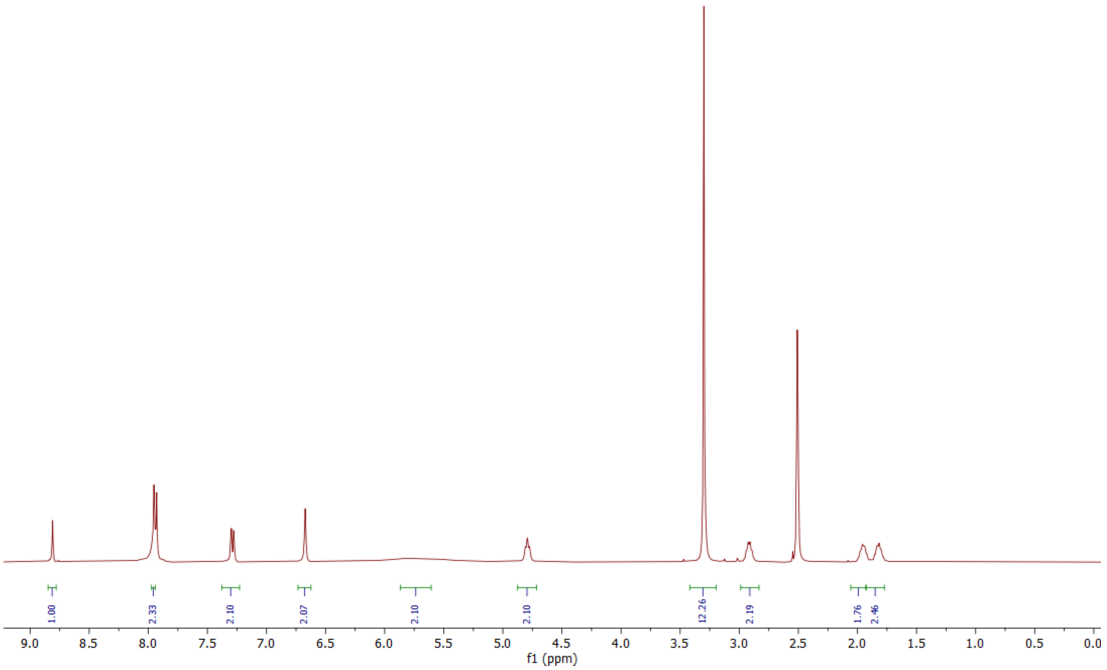

(e) ^{13}C NMR (400 MHz, DMSO-d_6) spectrum of St17

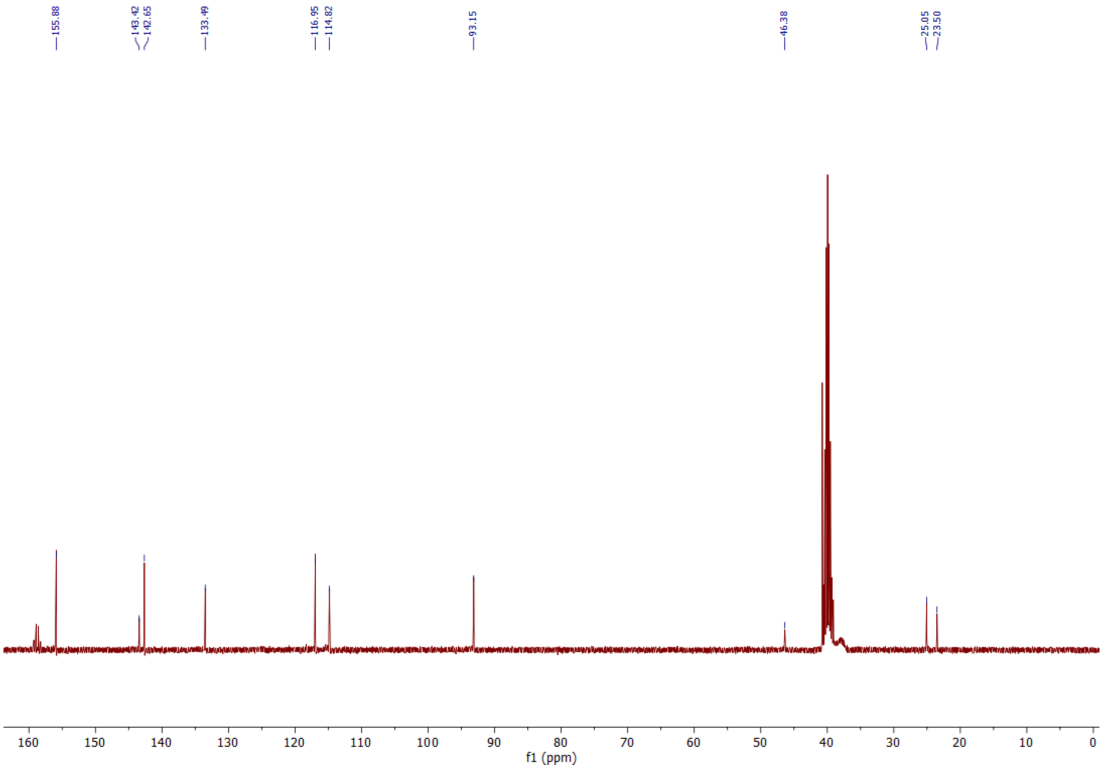

(f) ^{1}H NMR (400 MHz, DMSO-d_6) spectrum of Mal-Acridine Orange (**12**)

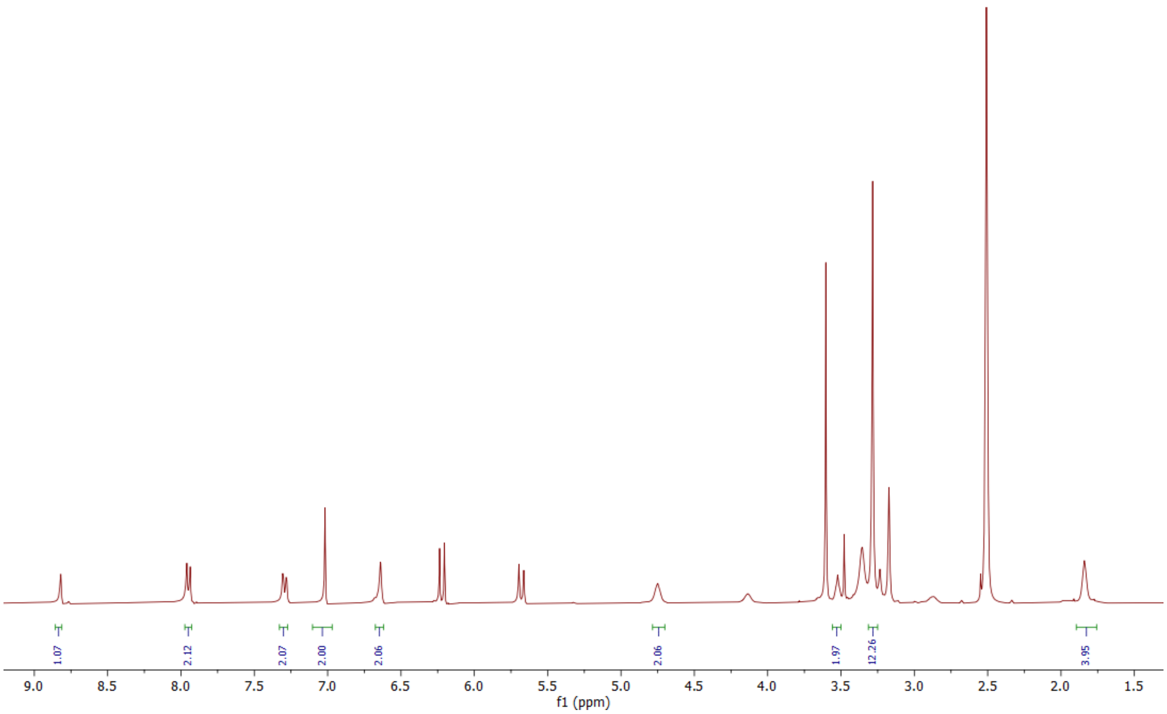

759 (g) ^{13}C NMR (400 MHz, DMSO-d_6) spectrum of Mal-Acridine Orange (**12**)

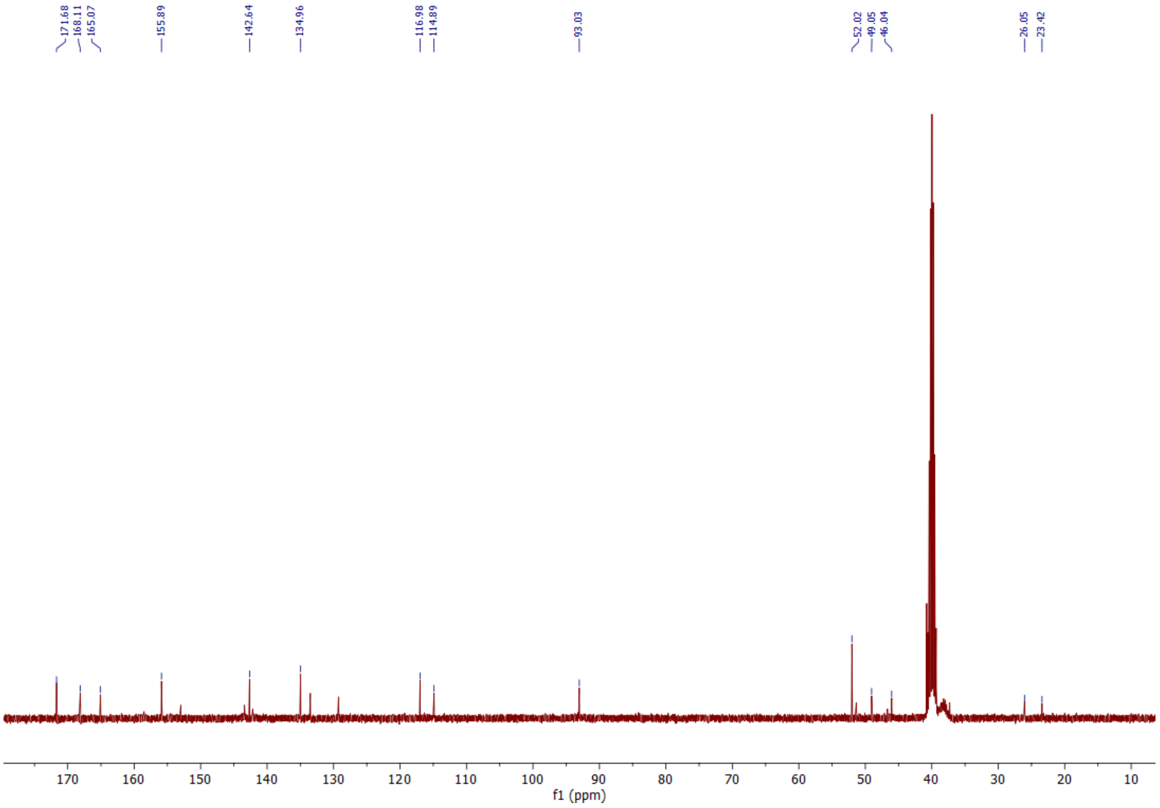

Figure S12. Characterization of HO-MOPs-AO.

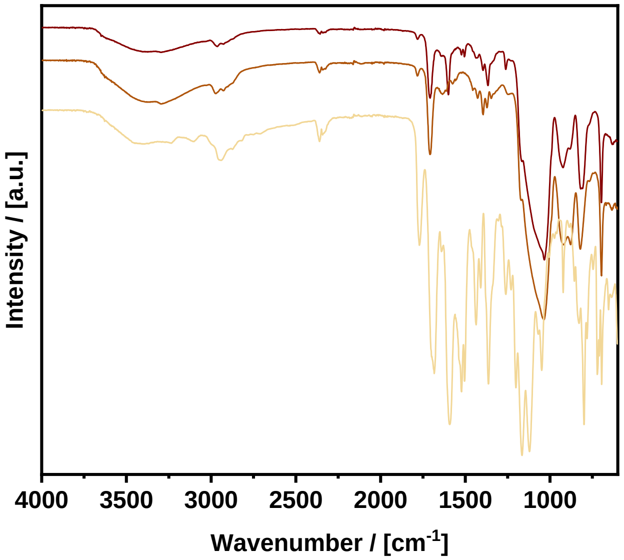

(a) ATR-IR

Dark brown: HO-MOPs-AO; Light brown: HO-MOPs-SH; Orange: Mal-Acridine Orange (12)

Assignment of bands:

Sample	Wavenumber / [cm^{-1}]	vibration
Light brown	1040	Si-O (nanoparticle)
	1710	C=O (maleimide)
	1455	Triazole (Hoechst)
	1615	Triazole (Hoechst)
Dark brown (only additional bands)	1600	Amine (Acridine Orange)

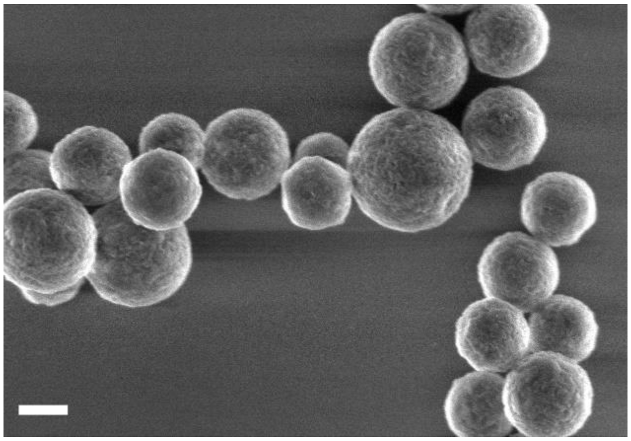

(b) SEM image HO-MOPs-AO nanoparticles; Scale bar represents 200 nm

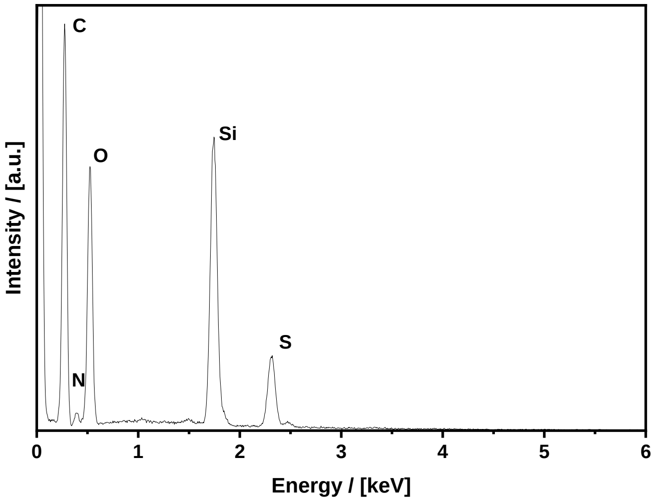

(c) EDX measurement of HO-MOPs-AO nanoparticles

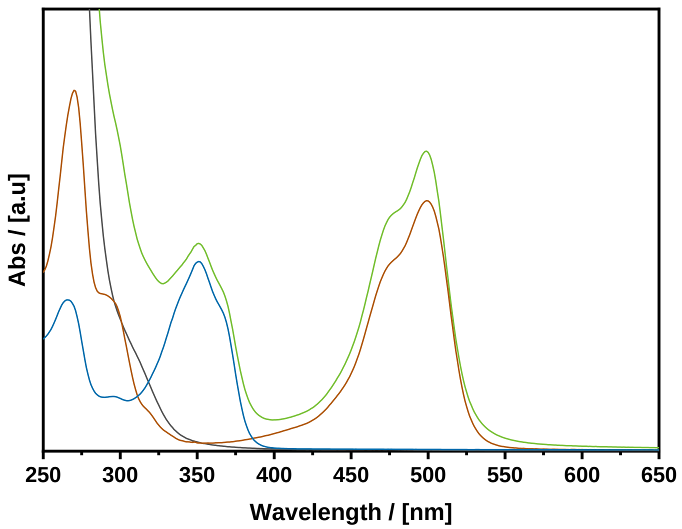

(d) UV-Vis measurement of HO-MOPs-AO nanoparticles and Mal-Acridine Orange

Black: YN-MOPs-SH; Green: HO-MOPs-AO; Dark blue: Hoechst-PEG-Azide (7); Brown: Mal-Acridine Orange (12)

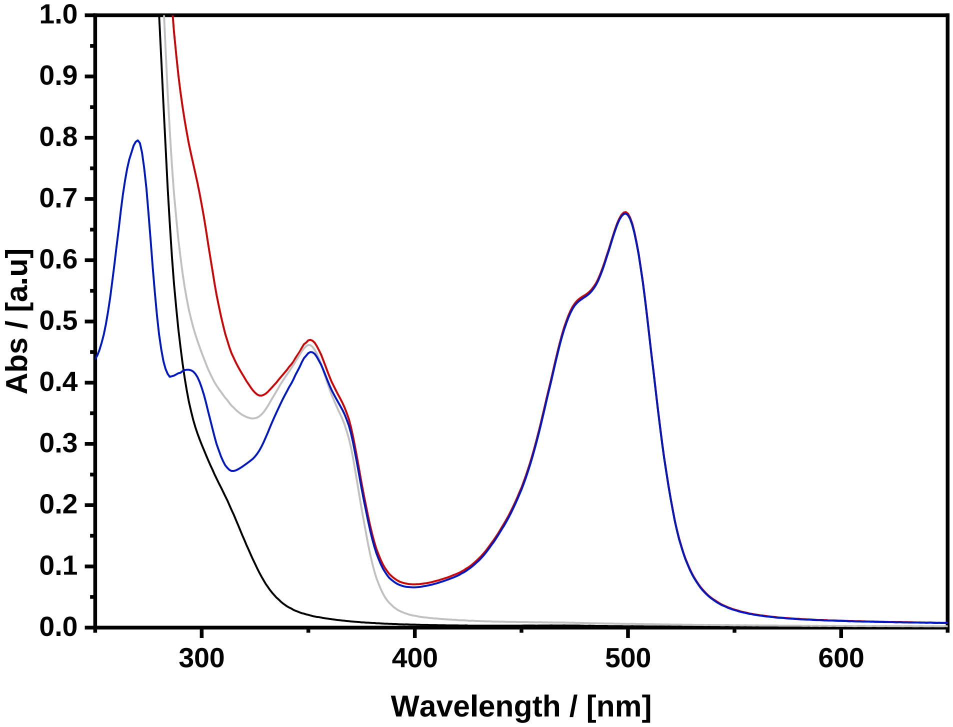

(e) Post-functionalization degree of HO-MOPs-AO nanoparticles using molar extinction coefficients calculated in f and g:

Black: absorption spectrum of dissolved MOPs-SH; Grey: absorption spectrum of dissolved HO-MOPs-SH; Red: absorption spectrum of dissolved HO-MOPs-AO; Blue: corrected absorption spectrum of dissolved HO-MOPs-AO (Correction was executed by subtracting the spectrum of MOPs-SH and HO-MOPs-SH from the spectrum of HO-MOPs-AO to exclude the signal intensity resulting from the MOPs-SH)

Hoechst-PEG-Azide (7)	Mal-Acridine Orange (12)
2 %	10 %

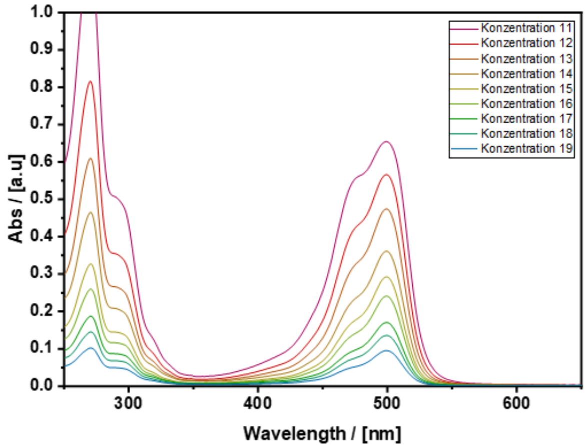

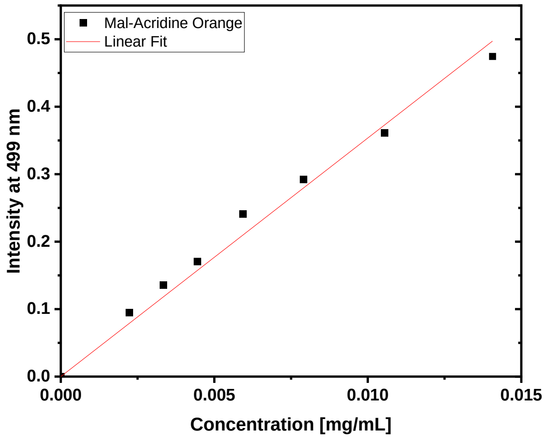

(f) Determination of molar extinction coefficient of Mal-Acridine Orange in 1m NaOH (90%) and DMSO (10%)

Calculation of molar extinction coefficient: ε = 35.3613 L*g-1*cm-1

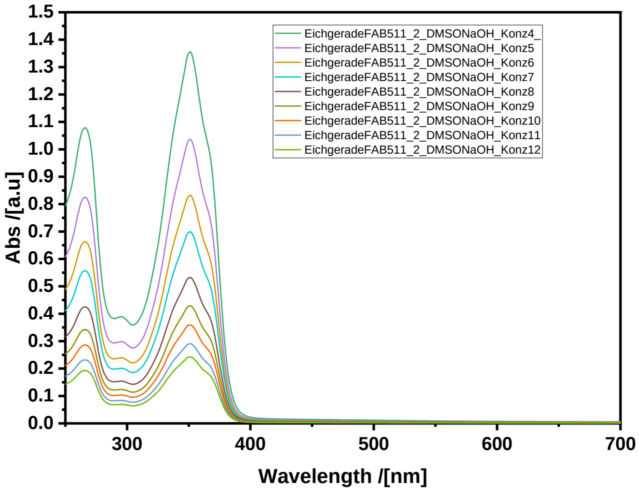

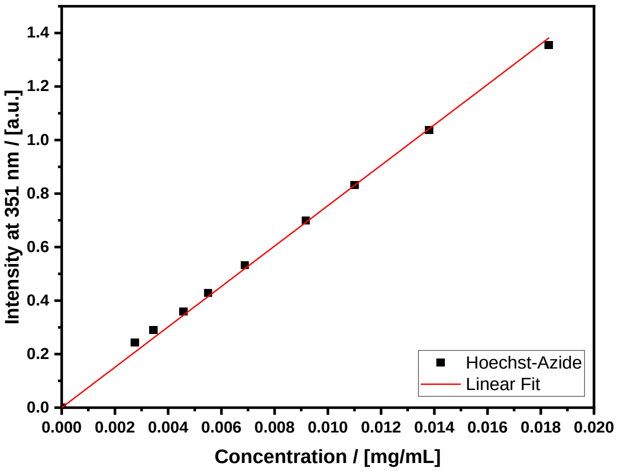

(g) Determination of molar extinction coefficient of Hoechst-PEG-Alk (7) in 1m NaOH (90%) and DMSO (10%)

Calculation of molar extinction coefficient: ε = 75.47615 L*g-1*cm-1

Figure S13. Excitation and emission spectrum of molecular Hoechst-PEG-Azide (7) and molecular Mal-Acridine Orange (12).

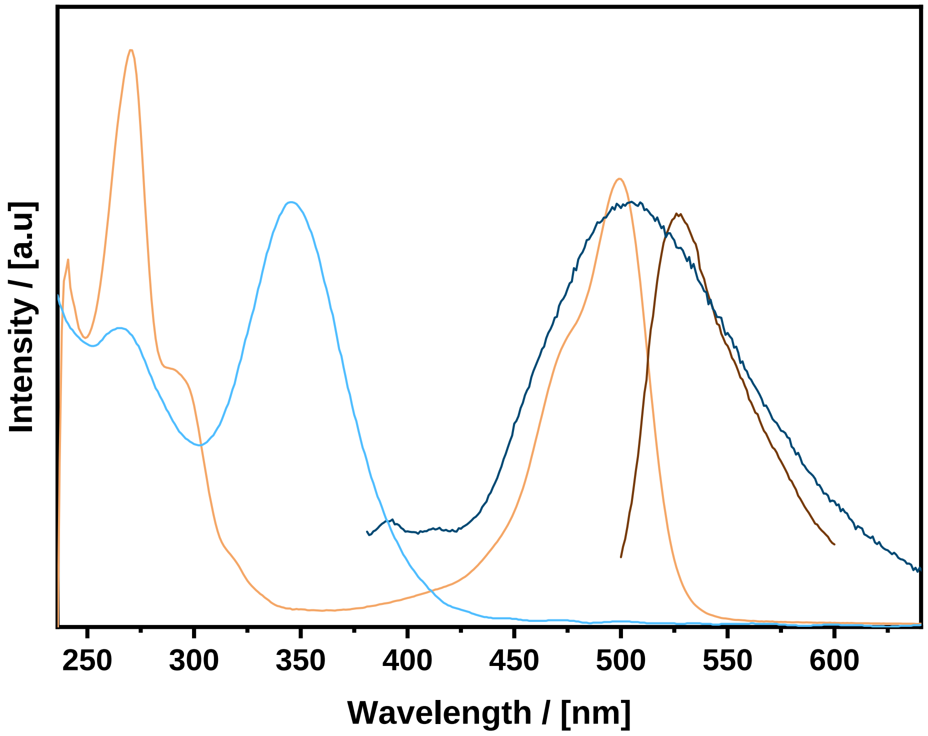

Light blue: Absorption of Hoechst-PEG-Azide (7)

Dark blue: Emission of Hoechst-PEG-Azide (7)

Light brown: Absorption of Mal-Acridine Orange (12)

Dark brown: Emission of Mal-Acridine Orange (12)

Table S1. Fluorescence measurements of HO-MOPs-AO nanoparticles and characterization of FRET.

Data for fluorescence lifetime measurements

(a): Fluorescent lifetimes τ, amplitudes A, amplitude weighted lifetimes $<\tau>$ and average lifetimes $\bar{\tau}$ resulting from the tri exponential reconvolution fits of the fluorescent decays measured as described above. The fluorescent decays were recorded at the emission wavelength λ_{FLT}.

	λ_{FLT} / nm	τ_1 / ns	τ_2 / ns	τ_3 / ns	A_1 / ns	A_2 / ns	A_3 / ns	$< \tau >$ / ns	$\bar{\tau}$ / ns
HO-MOPs-SH with 10 µg DNA	480	0.1436	0.8067	2.735	0.222	0.0724	0.0148	0.131	1.241
HO-MOPs-SH without DNA	490	0.2628	1.109	2.949	0.153	0.0832	0.0154	0.178	1.387
HO-MOPs-AO with 10 µg DNA	436	0.01605	2.365	6.695	1.331	0.0115	0.000567	0.052	1.720
HO-MOPs-AO without DNA	436	0.01152	0.1512	5.2	1.703	0.005311	0.000126	0.021	1.178

(b): FRET Efficiencies E_{FRET} calculated from the amplitude weighted lifetimes using equation (5).

$< \tau >_D$ from sample	$< \tau >_{DA}$ from sample	E_{FRET}
Ratio HO/AO 0.67/1		
HO-MOPs-SH	HO-MOPs-AO	88 %
HO-MOPs-AO with DNA	HO-MOPs-AO with DNA	60 %

Figure S14. Uric acid degradation under irradiation with a blue LED and without added nanoparticles; spectra were recorded after : 5, 15, 30, 45, 60, 75, 90 min; irradiation at 460 nm.

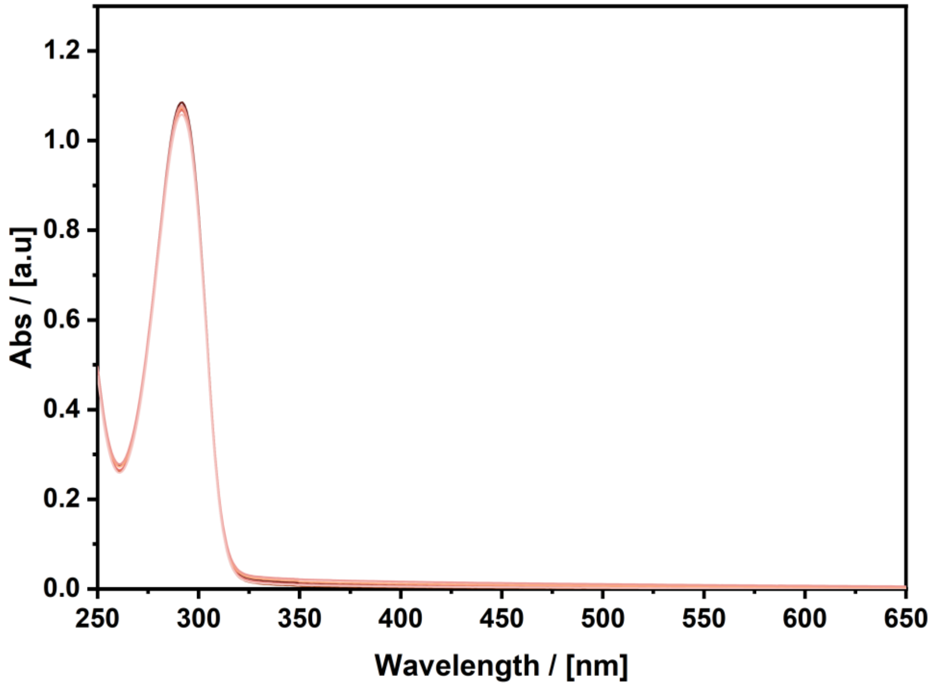

Figure S15. Singlet oxygen production of HO-MOPs-AO measured with ABDA (9,10-anthracenediyl-bis(methylene)dimalonic acid) degradation assay.

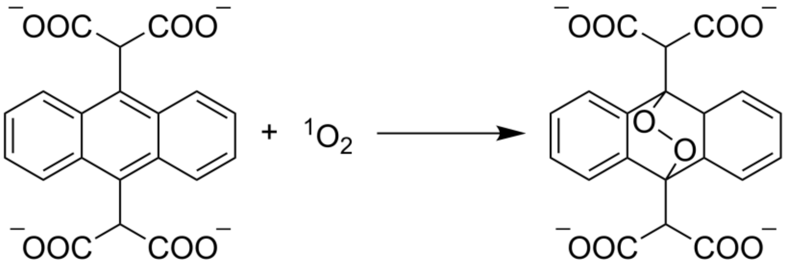

(a) Reaction of ABDA with singlet oxygen.[10]

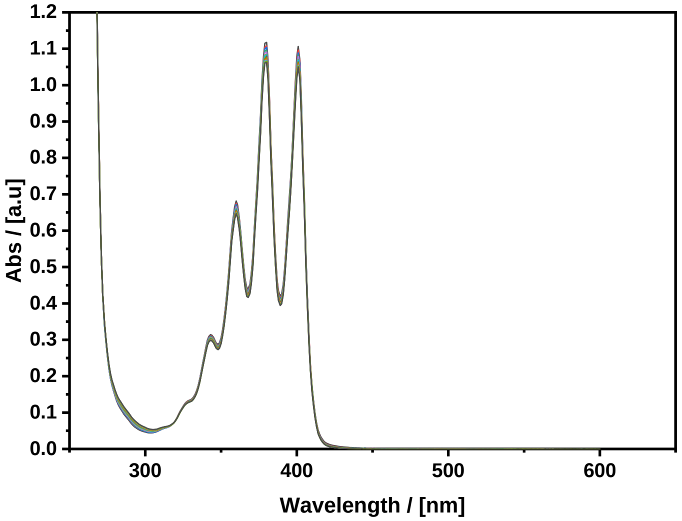

(b) ABDA degradation under irradiation with a blue LED and without added nanoparticles; spectra were recorded every 5 min for 1 h; irradiation at 460 nm.

Figure S16. Binding of MOPs to bare glass cover slides with the fluorescence of HO as an indicator; bright field image (left), DAPI filter image (middle), merged channels (right).

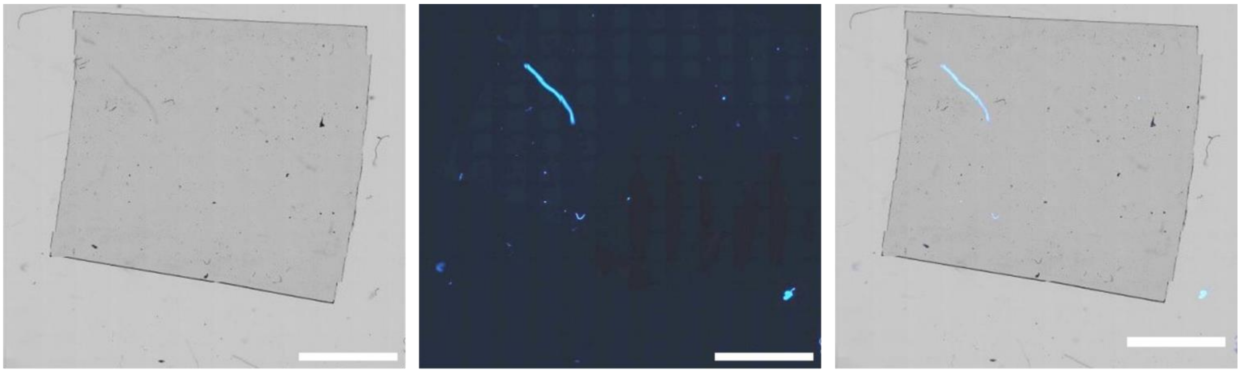

Figure S17. CFU counts after incubation and irradiation of *P. fluorescens* biofilms.

(a) Agar plate used for quantification of cell viability after irradiation with increasing amount of HO-MOPs-AO.

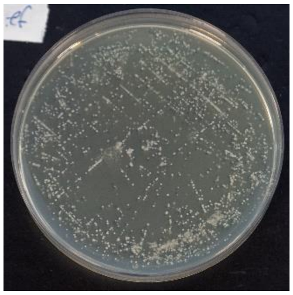

Ctrl not irradiated
Dillution 1/8000

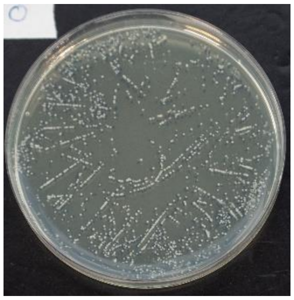

Irradiated with 0 µg/mL
nanoparticles
Dillution 1/8000

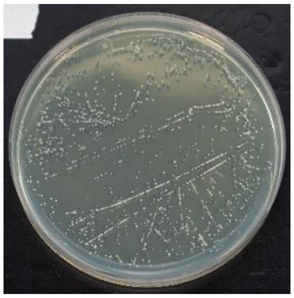

Irradiated with 10 µg/mL
nanoparticles
Dillution 1/8000

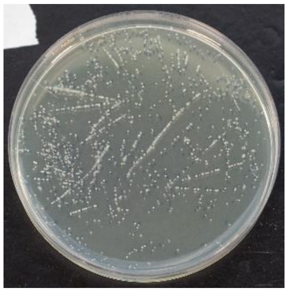

Irradiated with 50 µg/mL
nanoparticles
Dillution 1/8000

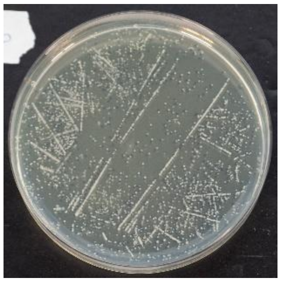

Irradiated with 100 µg/mL
nanoparticles
Dillution 1/1000

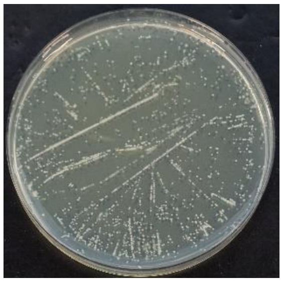

Irradiated with 250 µg/mL
nanoparticles
Dillution 1/1000

(b) Control experiments for cell viability of *P. fluorescens* in biofilm.
The biofilm was incubated with HO-MOPs-AO without irradiation (two left bars and agar plates below) and only with irradiation but without nanoparticles (two right bars).

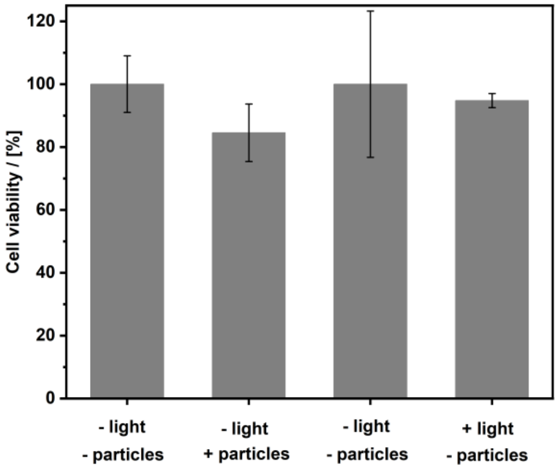

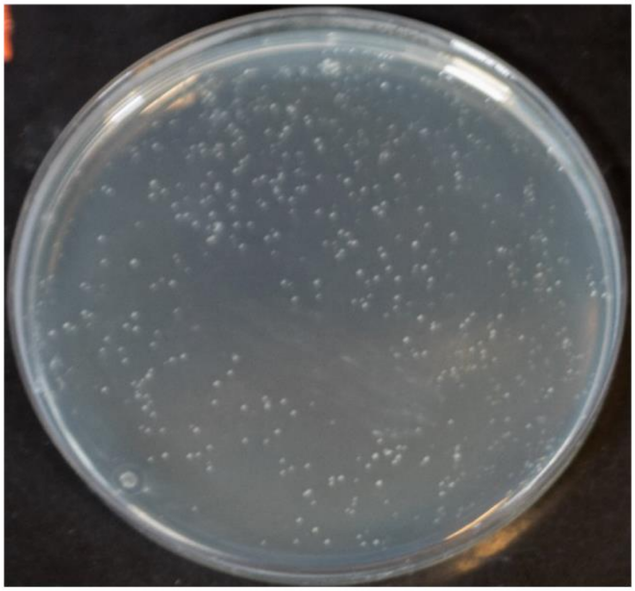

Ctrl without nanoparticles
Without irradiation

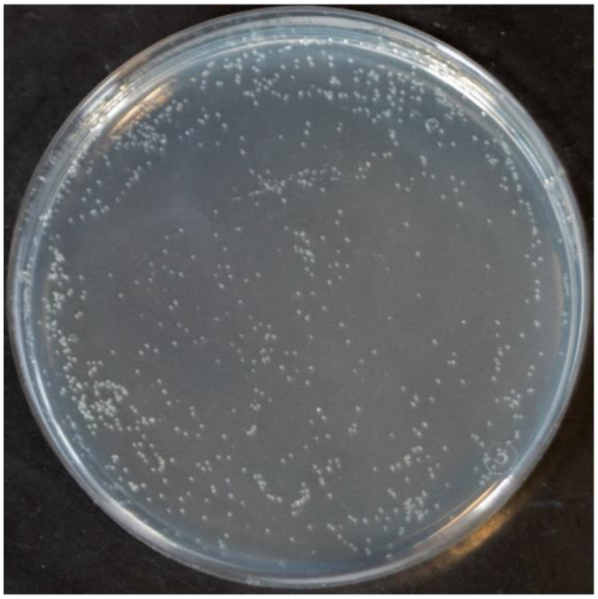

250 µg/mL nanoparticles
Without irradiation

References

(1) Gehring, J.; Schleheck, D.; Trepka, B.; Polarz, S. Mesoporous Organosilica Nanoparticles Containing Superacid and Click Functionalities Leading to Cooperativity in Biocidal Coatings. *ACS Appl. Mater. Interfaces* **2015**, *7* (1), 1021–1029. https://doi.org/10.1021/am5083057.

(2) Yan, R.; Sander, K.; Galante, E.; Rajkumar, V.; Badar, A.; Robson, M.; El-Emir, E.; Lythgoe, M. F.; Pedley, R. B.; Årstad, E. A One-Pot Three-Component Radiochemical Reaction for Rapid Assembly of ^{125}I-Labeled Molecular Probes. *J. Am. Chem. Soc.* **2013**, *135* (2), 703–709. https://doi.org/10.1021/ja307926g.

(3) Tuccitto, N.; Catania, G.; Pappalardo, A.; Trusso Sfrazzetto, G. Agile Detection of Chemical Warfare Agents by Machine Vision: A Supramolecular Approach. *Chem. – Eur. J.* **2021**, *27* (55), 13715–13718. https://doi.org/10.1002/chem.202102094.

(4) Wallace, K. J.; Morey, J.; Lynch, V. M.; Anslyn, E. V. Colorimetric Detection of Chemical Warfare Simulants. *New J. Chem.* **2005**, *29* (11), 1469–1474. https://doi.org/10.1039/B506100H.

(5) Sato, N.; Tsuji, G.; Sasaki, Y.; Usami, A.; Moki, T.; Onizuka, K.; Yamada, K.; Nagatsugi, F. A New Strategy for Site-Specific Alkylation of DNA Using Oligonucleotides Containing an Abasic Site and Alkylating Probes. *Chem. Commun.* **2015**, *51* (80), 14885–14888. https://doi.org/10.1039/C5CC03915K.

(6) Li, C.; Zhang, J.; Zhang, S.; Zhao, Y. Efficient Light-Harvesting Systems with Tunable Emission through Controlled Precipitation in Confined Nanospace. *Angew. Chem. Int. Ed.* **2019**, *58* (6), 1643–1647. https://doi.org/10.1002/anie.201812146.

(7) Zhang, H.; Li, X.; Shi, Q.; Li, Y.; Xia, G.; Chen, L.; Yang, Z.; Jiang, Z.-X. Highly Efficient Synthesis of Monodisperse Poly(Ethylene Glycols) and Derivatives through Macrocyclization of Oligo(Ethylene Glycols). *Angew. Chem. Int. Ed.* **2015**, *54* (12), 3763–3767. https://doi.org/10.1002/anie.201410309.

(8) Shen, H.; Liu, Z.; Zhang, P.; Tan, X.; Zhang, Z.; Li, C. Trifluoromethylation of Alkyl Radicals in Aqueous Solution. *J. Am. Chem. Soc.* **2017**, *139* (29), 9843–9846. https://doi.org/10.1021/jacs.7b06044.

(9) Li, H.; Zheng, Q.; Han, C. Click Synthesis of Podand Triazole -Linked Gold Nanoparticles as Highly Selective and Sensitive Colorimetric Probes for Lead(Ii) Ions. *Analyst* **2010**, *135* (6), 1360–1364. https://doi.org/10.1039/C0AN00023J.

(10) Entradas, T.; Waldron, S.; Volk, M. The Detection Sensitivity of Commonly Used Singlet Oxygen Probes in Aqueous Environments. *J. Photochem. Photobiol. B* **2020**, *204*, 111787. https://doi.org/10.1016/j.jphotobiol.2020.111787.
